# The clinical and molecular spectrum of *AGO2*-associated Lessel-Kreienkamp neurodevelopmental syndrome

**DOI:** 10.1186/s13073-026-01745-4

**Published:** 2026-08-22

**Authors:** Debora Tibbe, Christina Kiel, Olena Ielesicheva, Kerstin Robles de Maruri, Helia Mahboobi, Joschka Züghart, Hans-Hinrich Hönck, Christoph Meier, Fabiola Biasella, Marcela Legüe, María Francisca Lopez Avaria, Edward Blair, Tracy Lester, Benito Banos-Pinero, Jose S. Pulido, Adele Schneider, Rebecca Procopio, Chloe Quelin, Bailey J. Leal, Julian A. Martinez-Agosto, Stephanie A. Bottomley, Ágnes Till, Kinga Hadzsiev, Renata Szalai, Kathryn Nicole Weaver, Joel Fluss, Henri Margot, Berta Almoguera, Isabel Lorda-Sánchez, Lucía López-López, J. Austin Hamm, Himanshu Goel, Yasemin Alanay, Ozlem Akgun Doğan, Gulşah Şebnem Ozkose-Iyigel, Genevieve Baujat, Marion Lesieur-Sebellin, Sophie Rondeau, Katherine Schon, Joseph Christopher, Bertrand Isidor, Benjamin Cogne, Neena S. Agrawal, Ryan Dahlhauser, Yutaka Furuta, Rachel Rabin, John Pappas, Chirag Patel, Irma Järvelä, Merja Rauhala, Isabelle Schrauwen, Suzanne M. Leal, Siddharth Banka, Riya Tharakan, Céline Pebrel-Richard, Fanny Laffargue, Nelly Durand, Tristan Celse, Maja Hempel, Ilia Valentin, Andrea Gregorova, Lenka Noskova, Sara Baumgartner, Christa Überbacher, Kai Muru, Ülle Murumets, Stella Lilles, Katharina Steindl, Anita Rauch, Federica Ruscitti, Alain Verloes, Jonathan Levy, Joohyun Park, Tobias B. Haack, Ingrid Bader, Sophie Julia, Guillaume Banneau, Alison M. Muir, Davor Lessel, Hans-Jürgen Kreienkamp

**Affiliations:** 1 Institute of Human Genetics, University Medical Center Hamburg-Eppendorf, Martinistrasse 52, Hamburg, 20246 Germany; 2 Institute of Human Genetics, University of Regensburg, Regensburg, Germany; 3 Institute of Human Genetics, University Hospital Salzburg, Salzburg, Austria; 4 Section in Developmental Neurogenomics, Human Genetics Branch, National Institute of Mental Health, National Institutes of Health (NIH), Bethesda, MD USA; 5 Hospital Dr. Exequiel González Cortés, Santiago, Chile; 6 Facultad de Ciencias Médicas, Universidad de Santiago de Chile (USACH), Santiago, Chile; 7 Oxford Centre for Genomic Medicine, Oxford University Hospitals NHS Foundation Trust, Oxford, UK; 8 Oxford Genetics Laboratories, Oxford University Hospitals NHS Foundation Trust, The Churchill Hospital, Oxford, UK; 9 Retina Service, Wills Eye Hospital, 840 Walnut Street, Philadelphia, PA 19107 USA; 10 Department of Ophthalmology, Northwestern University, Feinberg School of Medicine, Chicago, IL 60611 USA; 11 Service de Génétique Clinique, CLAD Ouest, CHU Rennes, Hôpital Sud, Rennes, France; 12 Department of Human Genetics, University of California Los Angeles, Los Angeles, CA USA; 13 Department of Medical Genetics, Medical School, Clinical Centre, University of Pécs, Pécs, Hungary; 14 The Heart Institute and Division of Human Genetics, Cincinnati Children’s Hospital Medical Center, Cincinnati, USA; 15 Department of Pediatrics, University of Cincinnati College of Medicine, Cincinnati, USA; 16 Child Neurology Unit, Subspecialties Service, Geneva University Children’s Hospital, Geneva, Switzerland; 17 Genetic Medicine Division, Diagnostics Department, University Hospitals of Geneva, Geneva, Switzerland; 18 Department of Genetics and Genomics, Fundacion Jimenez Diaz University Hospital, Health Research Institute-Fundacion Jimenez Diaz, Universidad Autonoma de Madrid (IIS-FJD, UAM), Madrid, Spain; 19 Center for Biomedical Network Research On Rare Diseases (CIBERER), Madrid, Spain; 20 Pediatric Genetics, Dolly Parton Children’s Hospital, Knoxville, USA; 21 General Genetics Service, Hunter Genetics, Waratah, NSW Australia; 22 School of Medicine and Public Health, College of Health, Medicine and Wellbeing, University of Newcastle, Callaghan, NSW Australia; 23 Division of Pediatric Genetics, Department of Pediatrics, School of Medicine, Acibadem University, Istanbul, Türkiye; 24 Rare Diseases and Orphan Drugs Application and Research Center (ACURARE), Acibadem University, Istanbul, Türkiye; 25 Department of Genome Studies, Health Sciences Institute, Acibadem University, Istanbul, Türkiye; 26 Department of Translational Medicine, Health Sciences Institute, Acibadem University, Istanbul, Türkiye; 27 Service de Médecine Génomique des Maladies Rares et Institut Imagine UMR-1163 Inserm, Université Paris Cité, Hôpital Necker-Enfants Malades, Assistance Publique des Hôpitaux de Paris, Paris, France; 28 East Anglian Medical Genetics Service, Cambridge University Hospitals NHS Foundation Trust, Cambridge, UK; 29 Department of Genomic Medicine, University of Cambridge, Cambridge, UK; 30 Service de Génétique Médicale, CHU Nantes, 9 Quai Moncousu, Nantes Cedex 1, 44093 France; 31 L’Institut du Thorax, INSERM, CNRS, Université de Nantes, Nantes, 44007 France; 32 Department of Pediatrics, Division of Medical Genetics and Genomic Medicine, Vanderbilt University Medical Center, Nashville, TN USA; 33 Department of Pediatrics, New York University Grossman School of Medicine, New York, NY 10016 USA; 34 Genetic Health Queensland, Royal Brisbane & Women’s Hospital, Brisbane, QLD Australia; 35 Faculty of Health, Medicine and Behavioural Sciences, The University of Queensland, Brisbane, QLD Australia; 36 Department of Medical Genetics, University of Helsinki, Helsinki, Finland; 37 Disability Services, Wellbeing Services County of Kainuu, Kajaani, Finland; 38 Department of Translational Neurosciences, University of Arizona College of Medicine – Phoenix, Phoenix, AZ 85004 USA; 39 Center for Statistical Genetics, Gertrude H. Sergievsky Center, Department of Neurology, Columbia University Medical Centre, New York, NY 10032 USA; 40 Manchester Centre for Genomic Medicine, St Mary’s Hospital, Manchester University NHS Foundation Trust, Health Innovation Manchester, Manchester, UK; 41 Division of Evolution, Infection & Genomic Sciences, School of Biological Sciences, Faculty of Biology, Medicine and Health, University of Manchester, Manchester, UK; 42 Service de Cytogénétique Médicale, UIC CYTMRR, CHU Clermont-Ferrand, Clermont-Ferrand, France; 43 Service de Génétique Médicale, CHU de Clermont Ferrand, Clermont Ferrand, France; 44 Service de Génétique, Génomique Et Procréation, CHU Grenoble Alpes, Grenoble, France; 45 Institute of Human Genetics, University Heidelberg, Heidelberg, Germany; 46 Department of Medical Genetics, University Hospital Ostrava, Ostrava, Czech Republic; 47 Research Unit for Rare Diseases, Department of Pediatrics and Inherited Metabolic Disorders, 1st Faculty of Medicine, Charles University in Prague, Prague, Czech Republic; 48 Clinic for Pediatrics I, Medical University of Innsbruck, Innsbruck, Austria; 49 Institute for Human Genetics, Medical University Innsbruck, Innsbruck, Austria; 50 Institute of Clinical Medicine, University of Tartu, Tartu, Estonia; 51 Genetics and Personalized Medicine Clinic, Tartu University Hospital, Tartu, Estonia; 52 Children’s Clinic, Department of General Paediatrics and Neurology, Tartu University Hospital, Tartu, Estonia; 53 Institute of Medical Genetics, University of Zurich, Schlieren-Zurich, Switzerland; 54 Service de Génétique Clinique, Robert Debré - APHP Nord - Université Paris Cité, ERN-ITHACA, Paris, France; 55 Service de Cytogénomique, Hôpital Robert-Debré, APHP, Paris, France; 56 Institute of Medical Genetics and Applied Genomics, University of Tübingen, Tübingen, Germany; 57 Department of Clinical Genetics, CHU Toulouse, Toulouse, France; 58 GeneDx, LLC, Gaithersburg, MD USA; 59 Institute of Clinical Human Genetics, University Hospital Regensburg, Franz-Josef-Strauss-Allee 11, Regensburg, 93053 Germany

**Keywords:** RISC, RNA interference, P-bodies, IsomiR, GW182

## Abstract

**Background:**

Pathogenic variants in *AGO2*, encoding a central component of the RNA-induced silencing complex (RISC), cause the neurodevelopmental disorder Lessel-Kreienkamp syndrome (LESKRES). The variant spectrum and associated molecular mechanisms underlying phenotypic variability and disease severity remain incompletely understood.

**Methods:**

We investigated 45 newly identified individuals carrying 33 distinct *AGO2* variants, 30 of which were previously unreported. Phenotypic data from these and previously reported cases (*n* = 70) were integrated to delineate the LESKRES-associated clinical spectrum and genotype–phenotype correlations. Functional studies included shRNA-based silencing, co-immunoprecipitation, subcellular localization, and sequencing of AGO2-bound miRNAs.

**Results:**

All individuals presented with a neurodevelopmental disorder of variable severity. Delayed speech and language development (97%), intellectual disability (97%), and motor delay (93%) were the most consistent features, frequently accompanied by muscular hypotonia, autistic traits, attention deficit hyperactivity disorder, visual impairment and structural brain anomalies. Systemic manifestations, including skeletal, craniofacial, cardiac, and male urogenital anomalies were common, underscoring AGO2’s multisystemic role. Moreover, we report occurrence of gonadal mosaicism and reveal the presence of interfamilial and variant-specific clinical heterogeneity. Variants clustered in defined regions of AGO2, including the L1 loop, helix-7, and multiple loops of the PIWI domain, highlight structural hotspots critical for RISC activity. Not all pathogenic variants impaired shRNA-mediated silencing; this was restricted to p.(Arg714Trp) and p.(Asn729His). Biochemical analyses revealed that p.(Asp619Asn) impaired GW182 binding and P-body assembly. Variants p.(Arg506Gln), p.(Glu531Gln) p.(Gly604Arg) and p.(Asp619Asn), reduced C-terminal phosphorylation, implicating defective AGO2 recycling. AGO2–miRNA co-immunoprecipitation and sequencing demonstrated variant-specific perturbations in miRNA association, strand selectivity, and isomiR generation. Variants near the hinge of the helix-7 region, especially p.(Phe182del), induced extensive changes in miRNA association and 3′-end modification, suggesting impaired anchoring within the miRNA-binding pocket.

**Conclusions:**

Our findings substantially broaden the clinical and molecular landscape of LESKRES, establishing AGO2 as a pivotal regulator of neurodevelopment whose structural integrity is essential for precise miRNA-mediated gene regulation. Pathogenic variants disrupt distinct interconnected processes: P-body association, phosphorylation-dependent turnover, and miRNA interactions, culminating in dysregulated post-transcriptional gene silencing. These mechanistic insights link specific structural perturbations in AGO2 to graded clinical outcomes and underscore the critical role of AGO2 conformational dynamics in human neurodevelopment.

**Supplementary Information:**

The online version contains supplementary material available at 10.1186/s13073-026-01745-4.

## Background

RNA interference (RNAi) is essential for the posttranscriptional regulation of gene expression [1–3]. RNAi is driven by RNA induced silencing complexes (RISCs), which are formed by one of the several thousand microRNAs (miRNAs) bound to one of four Argonaute proteins (AGO1-4) [4, 5]. RISCs scan cellular mRNAs for sequences complementary to this “guide” RNA. This leads to translational silencing and degradation of the mRNA in processing (P-) bodies [6]. RISC regulated gene expression is crucial for neuronal differentiation and function [7, 8].

Pathogenic variants in *AGO1* and *AGO2* are associated with similar neurodevelopmental disorders [9, 10], termed Neurodevelopmental disorder with language delay and behavioral abnormalities, with or without seizures (NEDLBAS, OMIM #620292) and Lessel-Kreienkamp syndrome (LESKRES; OMIM #619149), respectively. Both syndromes are characterized by intellectual disability, delayed speech and motor development, and prevalence of several further neurological symptoms. However, there is extensive variability in the severity and range of clinical manifestations. Our initial functional characterization suggested that pathogenic *AGO2* variants either result in impaired RISC formation or affect the speed by which AGO2 mediates recognition and silencing of the target mRNA [9].

Further *in-depth* analyses of five *AGO2* variants highlighted distinct biochemical consequences that can be partially correlated with the severity of clinical symptoms. We suggested that specific variants within linker 1 (L1) and linker 2 (L2) domains disrupt AGO2 conformational dynamics, thereby compromising its control over miRNA–target interactions, resulting in aberrant regulation of mRNAs. Such *AGO2* variants result in gain-of-function, and cause more severe clinical outcomes compared to loss-of-function variants [11].

Despite extended functional characterization of some selected variants, the LESKRES clinical spectrum and genotype–phenotype correlations remain elusive.

Here we define the clinical and variant landscape of *AGO2*-associated LESKRES. We describe a cohort of 45 affected individuals, and 30 new disease-causing variants, 6 of which are recurrent, and compare the findings to 25 previously documented cases [9, 12]. Our functional analysis revealed multiple pathomechanisms involved in the aetiology of this syndrome. This includes impaired silencing activity, AGO2 phosphorylation, cellular localization, protein interactions and aberrant miRNA-binding.

## Methods

### Human subjects

This study includes a cohort of 45 affected individuals presenting with variable neurodevelopmental delay in whom a rare *AGO2* variant was identified either in a diagnostic or research setting. The individuals were recruited through GeneMatcher [13] and our network of collaborators. Some affected individuals were enrolled through the “Lessel-Kreienkamp syndrome parent support group”, https://www.facebook.com/groups/ago2lesselkreienkamp. In such cases, the families/legal representatives were asked to provide the contact details of attending physicians in order to obtain objective and accurate clinical and genetic data. In cases where communication with the attending physician was not feasible, clinical data were obtained through a structured interview. Written informed consent for all affected individuals was obtained from the parents or legal guardians in accordance with protocols approved by the respective ethics committees of the institutions involved in this study.

### Genetic analyses and variant classification

Next-generation sequencing was performed in various research or diagnostic laboratories worldwide, as described [9, 14, 15]. All identified *AGO2* variants were classified according to the 2015 American College of Medical Genetics and Genomics/Association for Molecular Pathology (ACMG/AMP) guidelines for sequence variant interpretation [16]. Additional guidance for variant interpretation and evidence strength assignment was obtained from the Association for Clinical Genomic Science (ACGS) Best practice guidelines for variant classification in rare disease 2024. Computational evidence (PP3/BP4) was refined according to ClinGen sequence variant interpretation (SVI) recommendations for PP3/BP4 criteria [17], utilizing BayesDel [18] as the primary in silico predictor. Functional evidence (PS3/BS3) was evaluated according to current ClinGen SVI recommendations for interpretation of functional assays [19].

### Plasmids

Expression vectors for Shank3-mRFP and GFP-δ-catenin have been described [20]. cDNA coding for residues D599-L683 of TNRC6B (human Gw182; NM_015088.3) was cloned into pGEX4T-1. An expression vector for GFP-tagged human AGO2 was obtained from Phil Sharp (MIT) via Addgene (#21981; [21]). The vector for Flag/HA tagged human AGO2 was obtained from Gunter Meister (Univ. of Regensburg, Germany) [22]. Variants were introduced using the QuikChange II kit (Agilent; CA), using complementary oligonucleotides. Constructs were verified by Sanger sequencing. For expression in neurons, cDNA fragments coding for GFP-AGO2 were subcloned into the FUW vector which uses an ubiquitin promoter (obtained via Addgene #14882 from D. Baltimore, Caltech [23]).

### Antibodies

The following primary antibodies were used: chicken anti-MAP2 (Antibodies Online; ICC: 1:500); rabbit anti-Dcp1A (Abcam #183709; ICC 1:1000); rat anti-mRFP (5F8; Chromotek; WB: 1:2000); rat anti-AGO2 (EMD Millipore; clone 11A9; #MABE253; WB: 1:1000); rabbit anti-DICER1 (Bethyl #A301-936A; WB: 1:1000); mouse anti-phospho-serine (clone A4A; Sigma Aldrich #05-1000; WB: 1:1000), rabbit anti-DDX6 (Novus Biological #NB200-192; ICC: 1:500). Secondary antibodies: Alexa fluor 633 goat anti-rabbit (Thermo Fisher Scientific; A-21072; ICC: 1:1000), Alexa 405 goat anti-chicken IgG (Abcam; ab175674; ICC: 1:1000), Alexa 555 goat anti-guinea pig (Thermo Fisher Scientific; A-21435 ICC: 1:1000 dilution).

### shRNA-based silencing assays

A derivative of the HEK293T cell line in which the endogenous *AGO2* gene has been deleted by CrispR-based gene editing has been described [9]. Here, shRNA-based gene silencing is no longer possible [24]. Cells were plated on 12 well dishes and transfected 4–6 h later using Turbofect (3 µl/well) with an expression vector for a gene of interest (Shank3-mRFP or GFP-tagged δ-catenin) in combination with an shRNA vector targeting the corresponding mRNA. shRNA constructs in pSuper for δ-catenin and in pLVTHM for Shank3 using target sequences GCAACTATGTCGACTTCTA (mouse δ-catenin; 4240—4258 in NM_008729.3) and GGAAGTCACCAGAGGACAAGA (rat Shank3; 3794–3814 in NM_021676.2) have been described [9]. Either a control vector, or *AGO2* vectors were added to the transfection mix (0.5 µg/well). Cells were lysed in RIPA buffer and analyzed by Western blotting for the encoded protein.

### Pulldown experiments with GST-GW182 fusion proteins

GST-GW182 fusion protein was expressed in the Bl21 E.coli strain; after induction of bacterial cultures with IPTG at 20 °C overnight, fusion protein was purified from bacterial lysates with glutathione sepharose beads (Cytiva, Freiburg, Germany) following established procedures [25]. Protein was left on the beads, and attachment of GST-GW182 protein to the beads was verified by SDS-PAGE.

For pulldown assays, HEK293T cells were transfected with GFP-*AGO2* expression vectors using Turbofect reagent. On the next day, cells were lysed in 1 ml of IP buffer (50 mM Tris–HCl, 120 mM NaCl, 0.5% NP-40, 1 mM EDTA, pH 8.0). Lysates were cleared by centrifugation at 20,000 × g/4°C. The supernatant was mixed with 40 µl of packed GST-GW182 loaded beads and incubated for one hour on a rotator. After washing (5 × with IP buffer), samples of lysate and bead-attached proteins were analyzed by SDS-PAGE and Western blotting using anti-GFP antibody. Blot signals were quantified by enhanced chemiluminescence on a ChemiDoc MP imaging system (BioRad), with ImageLab 6.1 software. Pulldown efficiency was determined as the ratio of precipitate to input signals.

### Immunoprecipitation of GFP-AGO2 variants

HEK293T cells expressing GFP-*AGO2* variants were lysed in IP buffer on ice, followed by centrifugation at 20,000 × g for 15 min at 4°C. GFP-tagged proteins were immunoprecipitated using 20 μl of magnetic GFP-trap beads (Chromotek, Munich). After incubation on a rotator for 1 h at 4 °C, beads were collected on a DynaMag2 magnetic stand (Invitrogen; 1 min on ice), followed by washing (5 × in IP buffer). Precipitates and samples from cell lysates were analyzed by Western blotting.

### Primary cultured neurons

Primary dissociated hippocampal neurons were isolated from embryonic (E18) rats, as described [20]. Neurons were transfected after 7 days in vitro (DIV7) with GFP-*AGO2* constructs generated in the FUW vector [9].

### Immunocytochemistry

HEK293T cells (one day after transfection) or neurons (DIV14; seven days after transfection) were fixed with 4% paraformaldehyde/sucrose in PBS and permeabilized with 0.1% Triton X-100 in PBS for 5 min at room temperature. After blocking (10% horse serum in PBS) for 1 h at room temperature, cells were incubated with primary antibodies for MAP2 (dendritic marker) and Dcp1a (P-body marker) overnight. HEK293T cells were stained for P-body markers Dcp1a or DDX6. Cells were washed in PBS, followed by 1 h of incubation with Alexa Fluor secondary antibodies. GFP-tagged proteins were visualized by the GFP fluorescence. Nuclei were stained with DAPI. The coverslips were mounted onto glass microscopic slides using ProLong™ Diamond Antifade mounting medium.

### Microscopy

Confocal images were acquired with a Leica Sp8 confocal microscope using a 63 × objective. Quantitative analysis of images was performed using ImageJ. Three independent experiments were performed for all imaging assays. For HEK293T-based experiments, AGO2-clusters and Dcp1a or DDX6 clusters were counted per cell and the number of clusters was normalized to the number of transfected cells. In neurons, AGO2-clusters were counted in cell bodies (somatic) and along entire dendritic branches (dendritic). Cluster density was obtained by dividing the number of AGO2 or Dcp1a clusters by µm of dendrite length. 15 neurons with a total of 45 dendrites per condition were evaluated.

### AGO2/miRNA immunoprecipitation

For isolation of AGO2-associated microRNAs, HEK293T cells were co-transfected with vectors coding for Flag/HA-tagged AGO2 variants and pEGFP-C1 using Turbofect transfection reagent. Efficient transfection was verified on the next day by fluorescence microscopy. After washing twice with cold PBS, cells were lysed for 15 min on ice in HEPES-buffer (10 mM HEPES, pH 7.3; 100 mM KCl; 0.5% NP-40; 5 mM MgCl_2_, 0.5 mM DTT; protease inhibitors PMSF, Aprotinin and Leupeptin, and recombinant RNAse inhibitors superasin and RNAsin [26]). Homogenates were centrifuged for 15 min at 20,000 × g, 4 °C to clear the lysate and a 100 μl sample of the supernatant was saved on ice as input sample. The remaining supernatant was incubated with 20 µl anti-HA coated magnetic beads (Pierce; #88836) at 4 °C for 2 h with rotation. Beads were collected on a DynaMag2 magnetic stand (Invitrogen; 1 min on ice) and the supernatant was aspirated. Beads were washed twice in low-salt buffer (50 mM Tris–HCl, pH 7.5; 1 mM MgCl_2_, 150 mM NaCl, 0.5% NP40, 0.5 mM DTT) and twice in high-salt buffer (adding 600 mM NaCl). After the last washing step, beads as well as input samples were resuspended in 700 µl QIAzol. After incubation for 5 min, 140 µl Chloroform were added, followed by vigorous shaking for 15 s and incubation for 3 min. Samples were centrifuged at 12,000 × g for 15 min at 4°C. The upper phase was mixed with 1.5 vol of ethanol, and RNA was isolated with the miRNeasy micro kit (Qiagen) following the manufacturer’s protocol.

### Small RNA sequencing

Small RNA libraries were prepared with the TruSeq Small RNA Library Prep Kit (Illumina). Sequencing was performed on an Illumina NextSeq 550 sequencer generating 36 bp single-end reads.

### Differential binding analysis of small RNA sequencing data

Differentially bound miRNAs were analyzed using the sRNAtoolbox suite [27]. Data were uploaded to the sRNAbench web application (version of December 2021; access on June 30, 2025) and processed with default parameters, except that quality filtering used the mean quality score with a Phred threshold of 20. Annotation was performed with the MirGeneDB 2.1 reference database [28] for *Homo sapiens*. To assess miRNA enrichment in IP versus Input samples, the percentage of miRNA-assigned reads relative to the total read count in the analysis per sample was used to account for differences in library size. To detect differentially bound miRNAs the sRNAde web application (access on June 30, 2025) with default parameters was used. Significantly differentially bound miRNAs were extracted from DESeq results (FDR < 0.05 and |log2 fold change|> 1; [29]). Visualization was performed in R version 4.2.3 using the ggplot2 package version 3.5.0.

### Analysis of miRNA strand ratios

Raw counts of mature miRNA strands were extracted from the sRNAbench output. Counts per million (CPM) were calculated for each strand using the edgeR package version 3.40.2 [30] in R. For downstream analyses, mature miRNAs strands were retained with > 1 CPM in at least two of three replicates in either the WT or variants. Only miRNAs for which both strands were detected were considered. If no passenger strand was assigned by the sRNAtoolbox annotation, the strand with the higher abundance was defined as the guide strand and the other strand as the passenger strand. For pairwise comparisons of variant versus WT, a linear model was fitted to the log2(passenger/guide) CPM ratio. Statistical significance was assessed at FDR < 0.05.

### Detection of isomiRs

Raw small RNA sequencing data were processed with the miRMaster 2.0 web application [31], using default settings. Further analyses of processed count data were performed in R. To reduce technical artifacts, a minimum count threshold of five reads per isomiR was applied. IsomiRs were categorized by their impact on the mature miRNA sequence, identifying trimming or extension at the 3′ or 5′ ends with or without sequence variations. An isomiR was considered unique to the variant if it was detected in at least two of three variant replicates and in at most one WT replicate. Differences in isomiR category distributions between variant and WT samples were tested using a chi-squared test, with statistical significance defined as *p* < 0.05.

## Results

### Molecular spectrum of *AGO2* variants

**Fig. 1:**
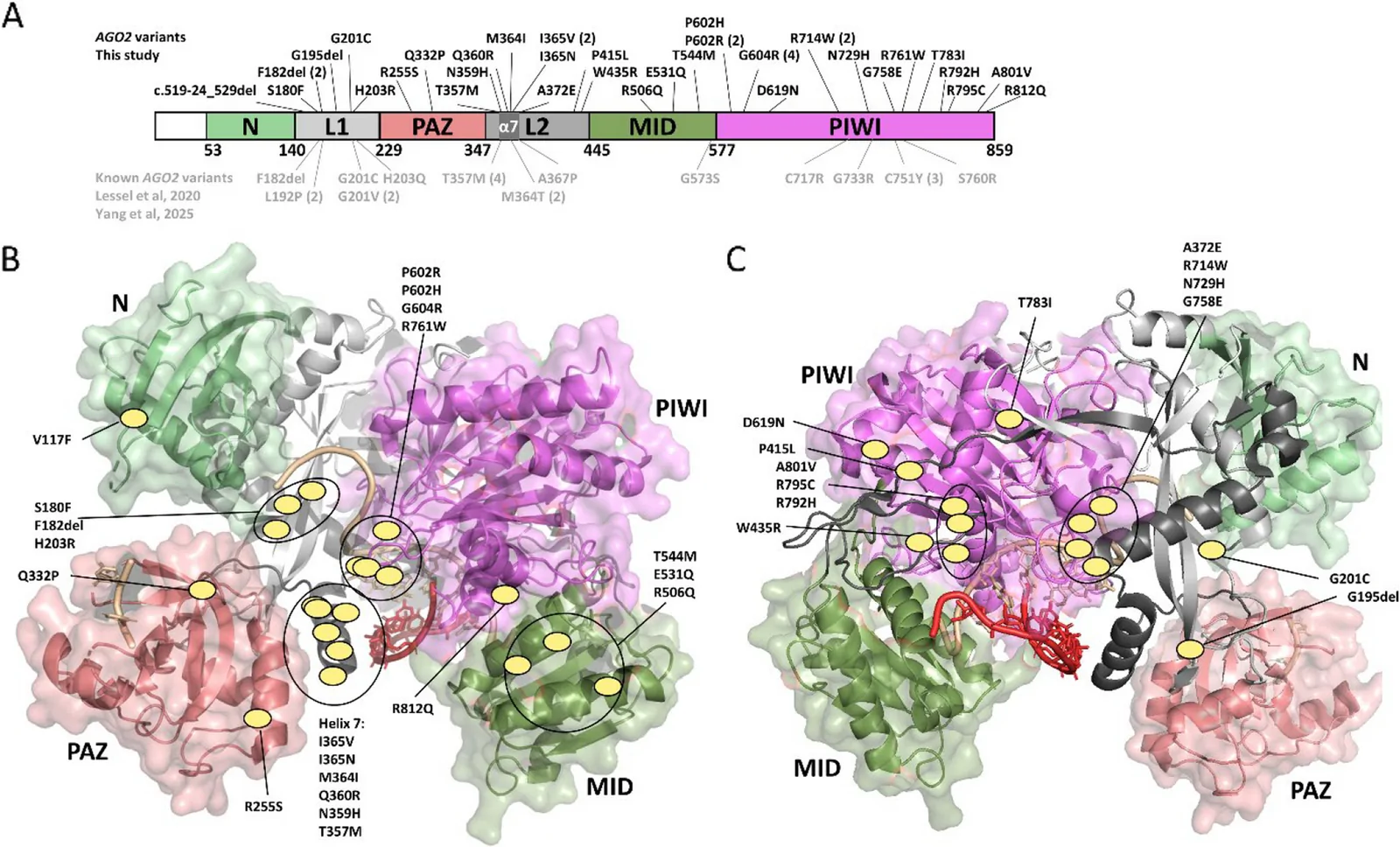
Positions of newly identified *AGO2* variants. **A** Domain structure of the AGO2 protein. New or recurrent variants identified in this manuscript are indicated in black print, previously identified variants are shown in gray. The recurrent variants are designated in parentheses. **B** 3D structure of human AGO2 in complex with a guide miRNA (colour: wheat) and a target mRNA (red). **C** The structure is rotated by 180°. AGO2 domains are coloured based on the scheme provided by Pymol: N, pale green; L1, light gray; PAZ, salmon; L2, dark gray; MID, smudge; and PIWI, violet. Positions of variants identified in this study are indicated by yellow circles

Here we describe 45 affected individuals harbouring a total of 33 *AGO2* (NM_012154.5) variants, 30 of which have not been reported before, and 6 are recurrent (Fig. 1). Out of the identified variants only the c.1517G > A, p.(Arg506Gln), recurrent variant c.1805C > G, p.(Pro602Arg), c.2383C > T, p.(Arg795Cys) and c.2402C > T, p.(Ala801Val) are present in the gnomAD dataset v4.1.0, however with allele frequencies of less than 0.000001 (Additional File 1: Table S1). The gross majority of variants occurred de novo. In individuals 6, 13, 24 and 31, harboring either the recurrent p.(Phe182del), p.(Asn359His), p.(Thr544Met) or the recurrent p.(Gly604Arg), respectively, parental testing was not possible. Individuals 2, 3 and 4 inherited the variant from the mildly affected mother (individual 1), similar to individual 11 who inherited the p.(Gln332Pro) from the mildly affected father. Individuals 29 and 30 (siblings) inherited the p.(Gly604Arg) from mosaic mother, similar to individual 43 who inherited the p.(Ala801Val) from mosaic father. Individual 38 inherited the p.(Thr783Ile) from the somewhat more mildly affected father (#39), and individual 40 inherited the p.(Arg792His) from the similarly affected mother. Notably, p.(Arg792His) was additionally identified in individual 41, in whom this variant occurred de novo. Collectively, the identified variants seem to cluster in specific parts of the encoded AGO2 protein (see Fig. 1 for an overview). The recurrent variants p.(Phe182del) and p.(Gly201Cys)/p.(Gly201Val), together with p.(Ser180Phe), p.(Gly195del) and p.(His203Arg)/p.(His203Gln), further emphasized the importance of the L1 loop, which was found to be a hotspot in our previous study [9]. Similarly, helix-7 in the L2 region contains another cluster of variants, recurrent p.(Thr357Met) and p.(Met364Thr)/p.Met364Ile), together with newly identified p.(Asn359His), p.(Gln360Arg), p.(Ile365Val) and p.(Ile365Asn) variants. We identified a novel hotspot region defined by p.(Pro602His) and recurrent variants p.(Pro602Arg) and p.(Gly604Arg). These residues reside on a loop (amino acids 602—608 in the PIWI domain; loop 1 in the nomenclature suggested by [4]) which is in direct contact with guide RNA nucleotide g9 in the structure of the AGO2 guide/target complex (Additional File 2: Fig. S1) [32]. This loop is required for tuning the strength of the seed guide/target interaction, possibly through interactions with a loop in the L2 region [33].

Variants p.(Arg714Trp), p.(Asn729His), p.(Gly758Glu) and p.(Arg761Trp) are located in other parts of the PIWI domain, particularly in loops 3_1_ and 3_2_ (Additional File 2: Fig. S2). Lastly, we observed clustering in loop 4 of the PIWI domain and an adjacent helix. Residues altered by variants p.(Arg792His), p.(Arg795Cys), p.(Ala801Val) and p.(Arg812Gln) are in contact with the guide/target RNA duplex (Additional File 2: Fig. S3). Beyond the missense variants, we identified a de novo 35 bp deletion (c.519-24_529del) in individual 45. This deletion spans the terminal 24 base pairs of intron 4 and the first 11 base pairs of exon 5, and is therefore expected to cause aberrant splicing, potentially resulting in a truncated protein product or causing haploinsufficiency. Taken together, according to ACMG guidelines [16] with additional refinement based on current ClinGen SVI and ACGS recommendations, all variants, apart from p.(Arg255Ser), p.(Asn359His) and p.(Arg761Trp), were classified as either likely pathogenic or pathogenic (Additional File 1: Table S1).

### Clinical spectrum of LESKRES individuals

All individuals harbouring LESKRES-associated *AGO2* variants presented with a neurodevelopmental disorder, albeit with variable expressivity and heterogeneity in clinical manifestations (Table 1 and Additional File 1: Table S1). Clinical information was available and analyzed for 70 affected individuals, comprising 45 newly reported cases and 25 from previous publications [9, 12]. Across the combined cohort, neurodevelopmental impairment was nearly universal, with delayed speech development (97%), intellectual disability (97%), and motor delay (93%) representing the most consistent and defining features of *AGO2*-associated LESKRES, followed by impaired receptive language in 81% of cases. While these features are clearly not specific to this disorder and are shared across a large number of Mendelian neurodevelopmental conditions, their high prevalence underscores their value as core components of the LESKRES phenotype. Consistent with earlier observations, the severity of intellectual impairment varied considerably, ranging from borderline (e.g., individual 31) to severe. Remarkably, individual 32, carrying the p.(Asp619Asn) variant, presented in the neonatal period with respiratory insufficiency, yet subsequently exhibited largely age-appropriate neurodevelopment.

**Table 1:** Summary of clinical signs and symptoms of individuals harboring pathogenic/likely pathogenic *AGO2* variants. Twenty-five patients from previous studies are included here [9, 12]

Clinical findings	this study	previous studies	all studies
Sex	14 female/31 male	15 female/10 male	29 female/40 male
Cognitive and motor development			
Impaired speech development	41/43	25/25	66/68 (97%)
Intellectual disability	40/42	25/25	65/67 (97%)
Motor developmental delay	39/44	25/25	64/69 (93%)
Impaired receptive language	30/41	17/17	47/58 (81%)
Dysmorphic features			
Epicanthic folds	18/38	15/25	33/63 (52%)
Thin upper lip	18/37	12/25	30/62 (48%)
Open mouth appearance	17/37	13/25	30/62 (48%)
Broad nasal bridge	19/36	7/25	26/61 (43%)
Deep set eyes	15/35	9/25	24/61 (39%)
Congenital craniofacial anomaly	13/37	11/25	24/62 (39%)
Upslanting palpebral fissures	12/37	10/25	22/62 (35%)
Frontal bossing	12/37	9/25	21/63 (33%)
Helix hypoplasia	9/35	6/25	15/60 (25%)
Dental anomalies	2/31	9/23	11/54 (20%)
Neurologic signs			
Muscular hypotonia	34/42	12/25	46/67 (69%)
Autistic features	27/42	9/20	36/62 (58%)
Attention deficit hyperactivity disorder	20/38	12/19	32/57 (56%)
Gait abnormalities	20/41	13/22	33/63 (52%)
Cerebral MRI abnormalities	13/33	9/17	22/50 (44%)
Seizures	13/44	8/22	21/66 (32%)
Agressive behaviour	15/42	4/21	19/63 (30%)
Respiratory abnormalities	15/42	5/23	20/65 (31%)
Visual impairment	11/44	7/25	18/69 (26%)
Myopia/Hyperopia	11/44	6/23	17/67 (25%)
Strabism	8/43	7/25	15/68 (22%)
Other findings			
Neonatal feeding difficulties	17/40	10/22	27/62 (44%)
Skeletal anomalies	18/44	9/23	27/67 (40%)
Gastrointestinal disorder	12/43	8/23	20/66 (30%)
Short stature	9/36	5/21	14/57 (25%)
Immune system anomalies	10/41	2/21	12/62 (19%)
Heart anomalies	6/44	6/21	12/65 (18%)
Congenital anomalies of the male urogenital tract	6/25 (male)	1/10 (male)	7/35 (20% male)

A broad spectrum of neurological features was observed across the cohort. Muscular hypotonia (69%), autistic traits (58%), attention deficit hyperactivity disorder (56%), gait abnormalities (52%), and structural brain abnormalities on MRI (44%) were among the most frequent findings. Seizures, aggressive behaviour, breathing abnormalities and visual impairment occurred in approximately one-third of individuals. Other recurrent findings included neonatal feeding difficulties (44%), skeletal anomalies (40%), gastrointestinal disorders (30%), short stature (25%) and heart anomalies (18%). Congenital anomalies of the male urogenital tract were identified in approximately one-fifth of affected males. Dysmorphic features were frequent but variable, most commonly including epicanthic folds (52%), thin upper lip (48%), open mouth appearance (48%) and congenital craniofacial anomalies (40%). The latter included plagiocephaly, retrognathia, micrognathia, macrocephaly and microcephaly. Noteworthy, we additionally documented progressive macrocephaly in eight and microcephaly in four affected individuals.

Collectively, these data underscore the multisystemic and phenotypically heterogeneous nature of the LESKRES-associated neurodevelopmental disorder.

### Analysis of shRNA-mediated silencing

We selected eight newly identified *AGO2* variants for further analyses in a broad array of functional assays. Additionally, we included the p.(Val117Phe) variant that was identified in 27 individuals, based on the gnomAD v4.1.0 dataset, and was thus considered a benign variant.

**Fig. 2:**
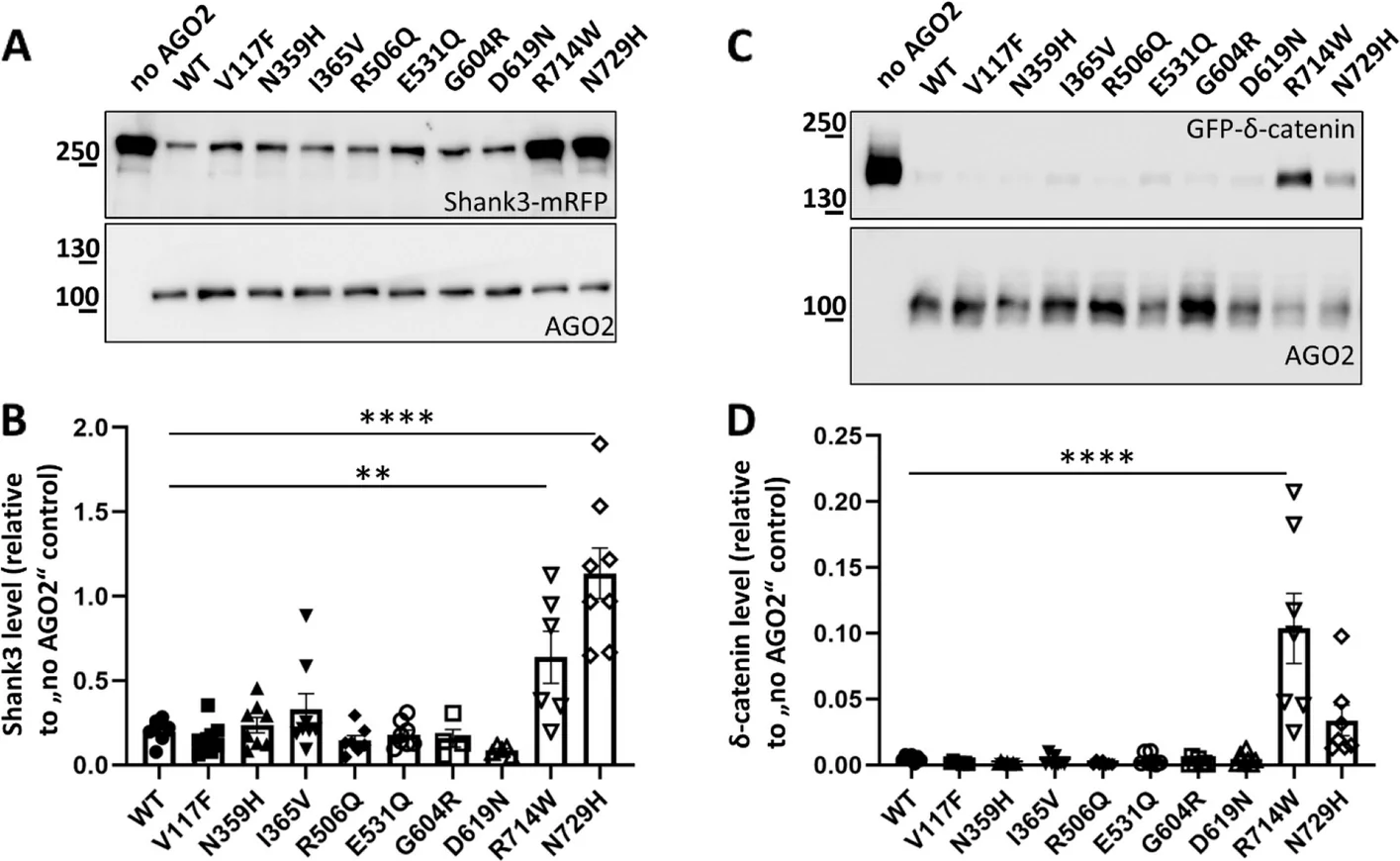
shRNA-based silencing assay. **A** *AGO2*-deficient HEK293T cells were transfected with a mix of plasmids coding for a rat Shank3-mRFP fusion, and an shRNA targeting Shank3. For each well, either empty vector, or vector coding for the indicated AGO2 variants were added. Cells were lysed and analyzed by Western blotting for the expressed proteins, as indicated. **B** Quantification of Shank3 blots shown in A. **, **** significantly different, *p* < 0.01, *p* < 0.0001; ANOVA, followed by Dunnett’s multiple comparisons test. **C**, **D** The assay was repeated for a pair of expression vectors coding for GFP-tagged δ-catenin and an shRNA against δ-catenin. ****, significantly different, *p* < 0.0001; ANOVA, followed by Dunnett’s multiple comparisons test (*N* = 7–8)

First, we performed shRNA-based assays in an AGO2-deficient derivative of the HEK293T cell line, as described [9]. Because shRNA-induced gene silencing is strictly dependent on AGO2 activity [24], we assessed AGO2 function by rescuing the silencing defect through expression of wild-type (WT) or mutant AGO2 constructs [9]. We used two different pairs of target mRNA/shRNA vectors: Shank3-shRNA/Shank3-mRFP and δ-catenin-shRNA/GFP-δ-catenin. For Shank3, we observed strong expression when the shRNA was present in AGO2-deficent cells. Ectopic expression of WT AGO2 strongly reduced expression of Shank3 to about 20—30% of control levels, confirming our previous findings [9] and the essential role of AGO2 in shRNA-based silencing. In contrast to our previous study, in which all analyzed LESKRES-associated *AGO2* variants exhibited impaired shRNA-mediated silencing [9], only two of the tested variants, p.(Arg714Trp) and p.(Asn729His) showed a deficit in this assay (Fig. 2A-B). Similarly, using the δ-catenin-shRNA/GFP-δ-catenin pair of vectors resulted in high expression of the expressed protein in the absence of AGO2. Again, WT-AGO2, could almost completely suppress expression of δ-catenin. p.(Arg714Trp) and p.(Asn729His) were again the only variants who showed less efficient suppression, with the difference being statistically significant for p.(Arg714Trp) (Fig. 2 C-D).

### Analysis of AGO2 interactions

**Fig. 3:**
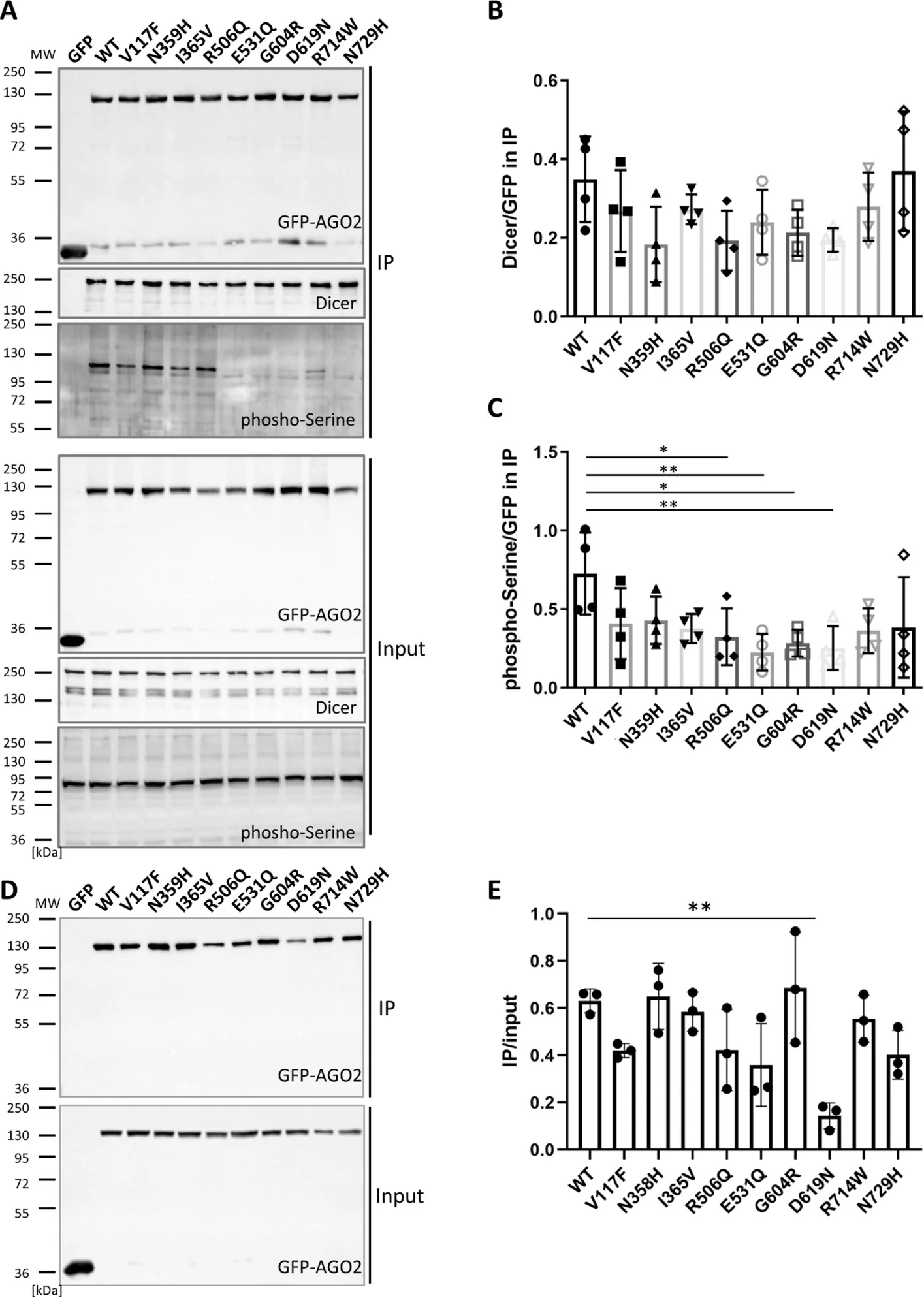
Co-immunoprecipitation analysis. **A** HEK293T cells were transfected with plasmids coding for GFP-tagged *AGO2* variants, or GFP-alone. GFP-tagged proteins were isolated from cell lysates by immunoprecipitation using the GFP-trap matrix, and input as well as precipitate samples were analyzed by Western blotting using anti-GFP, anti-Dicer and anti-phospho-Serine antibodies, as indicated. **B**, **C** Quantification of the data shown in A. Dicer (**B**) or phospho-Serine (**C**) signals in IP samples were normalized to GFP signals in IP samples. **D** A GST fusion protein carrying a fragment of the GW182 sequence involved in binding AGO2 was purified and left on GST-sepharose beads. Lysates from HEK293T cells expressing GFP-tagged AGO2 variants were prepared (input samples) and subjected to a pull down assay using the GST-GW182 sepharose beads (IP samples). GFP-tagged proteins in input and IP samples were analyzed by Western blotting. **E** Quantification of the data shown in D. *, ** significantly different from WT, *p* < 0.05, *p* < 0.01; ANOVA, followed by Dunnett’s multiple comparisons test, (*N* = 3–4)

In the next step, we performed protein–protein interaction profiling of AGO2 mutants. DICER participates in the biogenesis of miRNAs [34]. Argonaute proteins interact with DICER for efficient miRNA loading and RISC formation. We expressed GFP-AGO2 variants in HEK293T cells and immunoprecipitated the expressed proteins using GFP-trap. We observed specific and efficient co-precipitation of the endogenous DICER protein. However, no differences were observed between WT-AGO2 and any of the mutant proteins, indicating that the analyzed variants don’t interfere with this interaction (Fig. 3B).

In our previous study, we had observed that several pathogenic variants in *AGO2* resulted in reduced phosphorylation of a C-terminal cluster of four serine residues in AGO2 which is relevant for release of mRNA targets during RNA interference [9, 35, 36]. Therefore, we analyzed the immunoprecipitated GFP-AGO2 proteins for phosphorylation at serine residues using a phospho-Serine specific antibody. Phosphorylation of AGO2 was significantly reduced for p.(Arg506Gln), p.(Glu531Gln), p.(Gly604Arg) and p.(Asp619Asn) variants (Fig. 3C).

Processing (P-) bodies are cytoplasmic granules involved in the degradation of mRNAs as part of the RNAi pathway [6]. They are assembled through scaffolding interactions of TNRC6/GW182 protein family members [37–39]. Tryptophan residues in TNRC6/GW182 bind to specific pockets on the surface of the AGO2 PIWI domain, thereby recruiting AGO2 protein into the P-body complex [37, 40]. Our previous studies indicated that variants failing to localize to P-bodies and exhibiting impaired interaction with TNRC6/GW182 lead to a loss-of-function associated with a comparatively milder clinical presentation [9, 11]. We performed pulldown assays using a bacterially expressed GW182 fusion protein (Fig. 3D,E). These experiments revealed that the p.(Asp619Asn) variant exhibited reduced binding affinity for GW182, indicating a compromised ability to associate with P-body scaffolding components.

### Subcellular localisation of *AGO2* variants

**Fig. 4:**
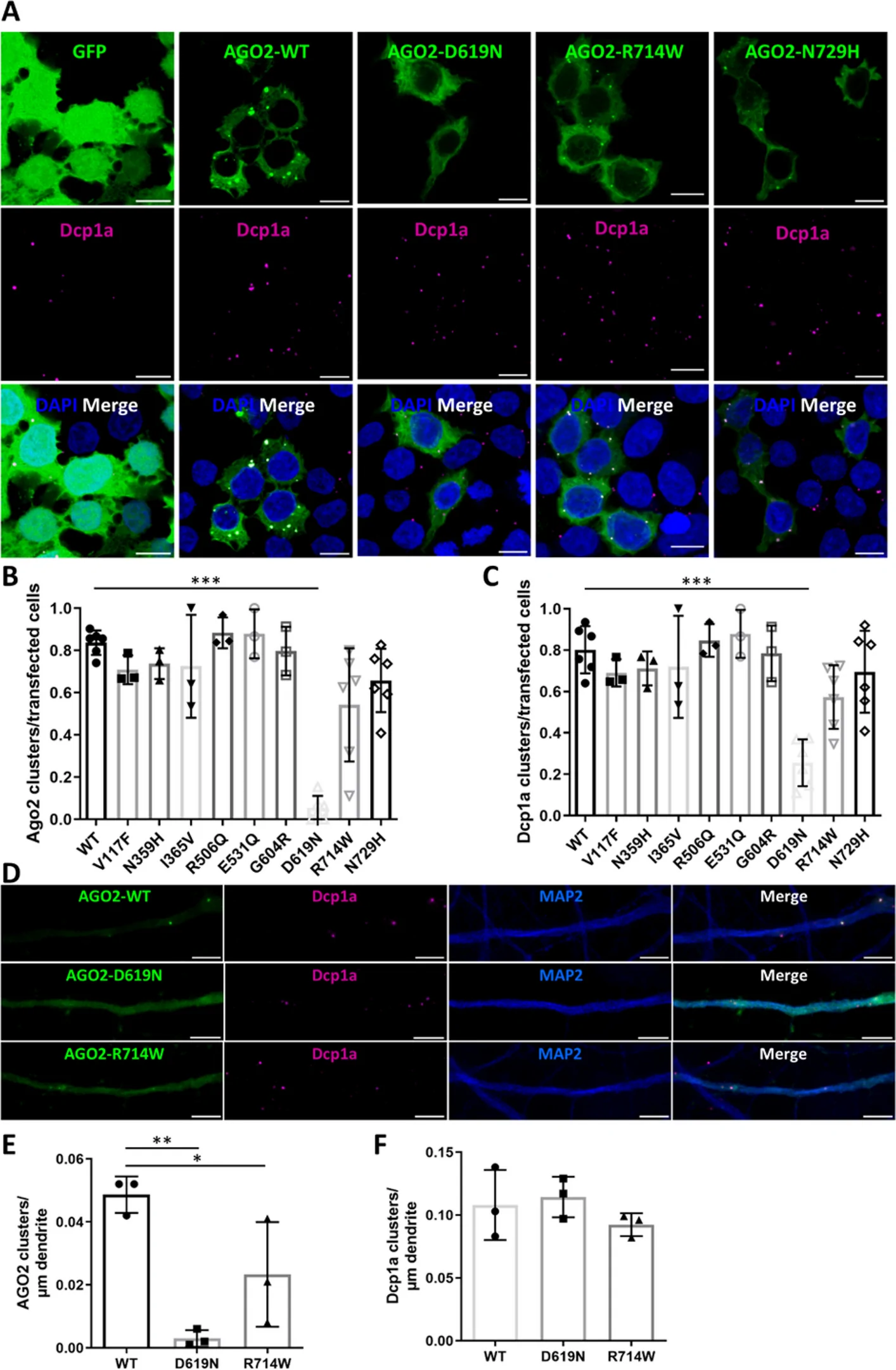
Cellular localisation of AGO2-mutants. **A** HEK293T cells transfected with plasmids coding for GFP or GFP-tagged AGO2 variants were fixed and stained for Dcp1a (magenta) and nuclear DNA (blue). Cells were analyzed by confocal microscopy. **B**, **C** Quantitative analysis of the number of GFP-AGO2 clusters (**B**) or Dcp1a clusters (**C**) normalized to the number of transfected cells. Cells from Additional File 2: Fig. S4 are included here. ***, significantly different from WT, < 0.001; ANOVA, followed by Dunnett’s multiple comparisons test. **D**-**F** Primary neurons expressing GFP-AGO2 variants were fixed and stained as in Additional File 2: Fig. S6. Dendritic segments were analyzed for the density of AGO2 and Dcp1a clusters normalized to the length of the dendritic segments. *, **, significantly different from WT, *p* < 0.05, < 0.01, respectively; ANOVA, followed by Dunnett’s multiple comparisons test (*n* = 3)

Based on these results, we asked whether the patient variants alter targeting of the AGO2 protein to P-bodies. In HEK293T cells, wild-type GFP–AGO2 formed distinct cytoplasmic clusters that colocalized with the P-body marker Dcp1A, whereas GFP alone exhibited a diffuse cytoplasmic distribution (Fig. 4A; Additional File 2: Fig. S4). Most *AGO2* variants tested also localized to Dcp1a-positive clusters, indicating preserved P-body association. In contrast, the p.(Asp619Asn) variant, consistent with its reduced binding to TNRC6/GW182, failed to localize to P-bodies. Other variants such as p.(Arg714Trp) showed reduced targeting to P-bodies, which however did not become statistically significant upon quantitative analysis (Fig. 4B). Strikingly, in cells expressing the p.(Asp619Asn) variant, Dcp1a-positive clusters were reduced or entirely absent, implying an impairment of P-body assembly (Fig. 4C). To confirm that this effect was not limited to a single P-body marker, cells expressing the p.(Asp619Asn) variant and a few other variants were additionally stained for DDX6, another well-established P-body component (Additional File 2: Fig. S5A). Again, cluster formation was strongly diminished for the p.(Asp619Asn) variant, demonstrating that this variant disrupts P-body formation rather than merely displacing individual components (Additional File 2: Fig. S5B and C).

Additionally, we analyzed the subcellular distribution of AGO2 variants in cultured hippocampal neurons (Fig. 4; Additional File 2: Fig. S6). Here, both p.(Asp619Asn) and p.(Arg714Trp) exhibited a reduced capacity to form P-body–like clusters within both the neuronal soma and dendritic compartments. However, in this cellular model, expression of the p.(Asp619Asn) variant did not alter the total number of P-bodies, as indicated by the unaltered abundance of Dcp1a-positive clusters (Additional File 2: Fig. S6C and S6D). The AGO2 variants analyzed here behave differently from previously analyzed variants p.(Phe182del), p.(Leu192Pro) and p.(Met364Thr), which induced an increase in the number of dendritic P-bodies [9, 11]. On the other hand, we have previously observed that the variant p.(Gly733Arg) (which appears completely non-functional as it binds neither to miRNAs nor to mRNA targets) fails to localize to P-bodies both in HEK293T cells and in neurons [9, 11]. As both p.(Gly733Arg) and p.(Asp619Asn) are associated with a particularly mild phenotype, the behaviour of AGO2 variants with respect to P-bodies may be helpful for further categorization of variants, as we have recently suggested [11].

Expression of *AGO2* variants did not affect neuronal morphology, as reflected by the number of MAP2-positive primary dendrites per neuron (Additional File 2: Fig. S4C).

### Altered association of mutant AGO2 protein with miRNAs

Next, we aimed to determine if and to which extent some of the identified variants affect the association of AGO2 with cellular miRNAs. Here we selected, as a positive control, the previously identified, recurrent p.(Phe182del) variant [9]. This variant is located in the hinge that enables helix-7 movements and affects the binding affinities of the AGO2-miRNA complexes to a target RNA [11]. The corresponding *AGO1* variant, p.(Phe180del) has been identified as the most frequent patient variant in the *AGO1*-associated NEDLBAS syndrome [10]. Introduction of this variant into the *C. elegans alg-1* gene leads to altered miRNA association and altered strand selectivity [41]. In addition, we included the p.(Asp619Asn) variant, resulting in the mildest clinical phenotype, and p.(Gly604Arg), representing the newly identified hotspot region within loop 1 of the PIWI domain. Moreover, p.(Asn359His), the only variant classified as variant of unknown significance in our cohort, was included with the aim to finally provide evidence for pathogenicity.

We expressed Flag/HA-tagged *AGO2* variants in HEK293T cells, immunoprecipitated AGO2-miRNA complexes using the anti-HA antibody and performed next-generation sequencing. Comparative analysis of miRNA profiles from cell lysates (input) and immunoprecipitates (IP) revealed a marked reduction in miRNA abundance in precipitates from cells transfected with the Flag/HA-empty vector relative to WT-AGO2, thereby validating the specificity of our approach (Fig. S7A). Notably, p.(Gly604Arg) bound fewer miRNAs when compared to WT-AGO2 (Additional File 2: Fig. S7A). A hierarchical clustering dendrogram was generated to evaluate the similarity between the input and IP sample datasets (Additional File 2: Fig. S7B).

**Fig. 5:**
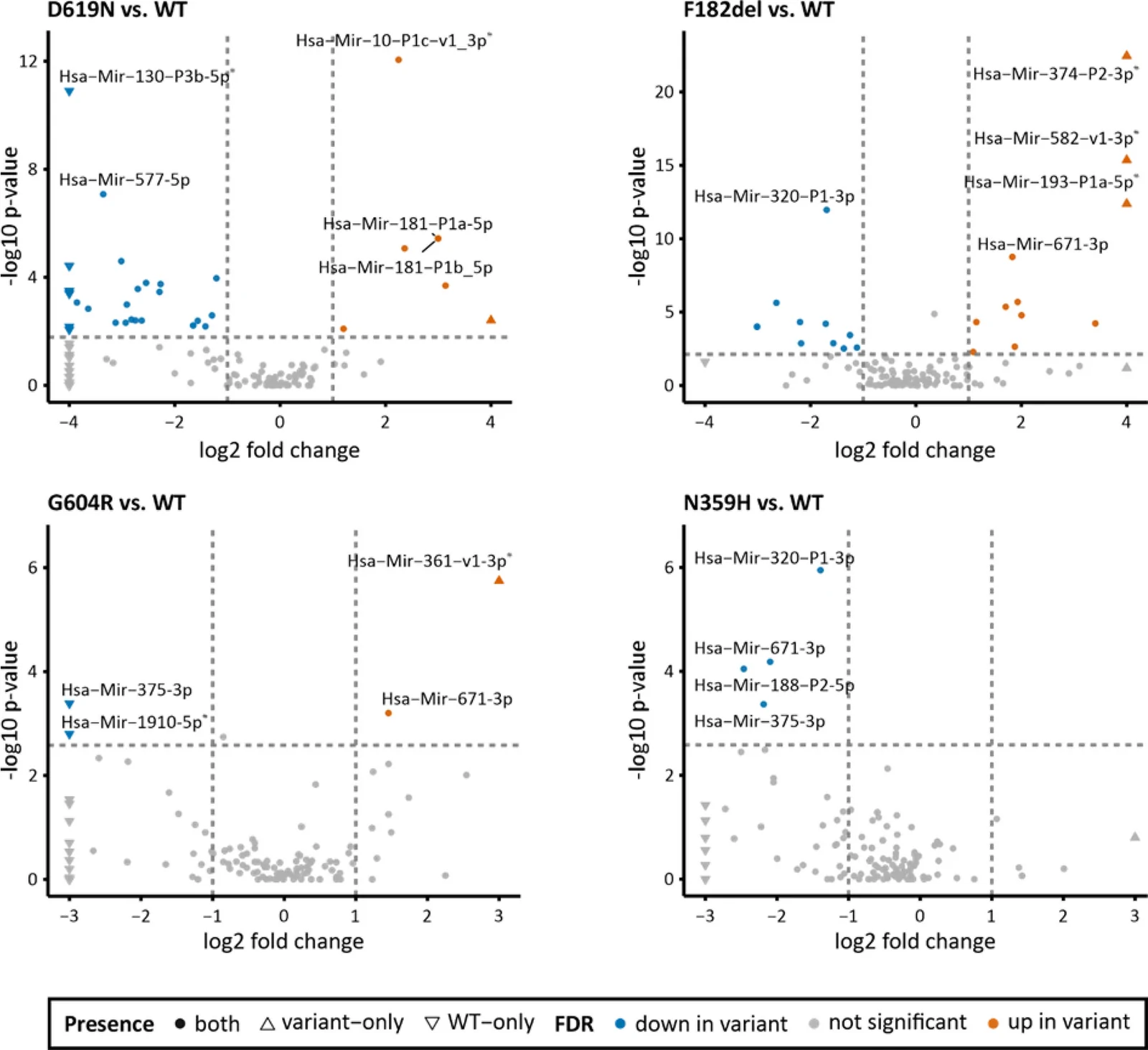
Differentially AGO2-bound miRNAs. Volcano plots of differentially bound miRNAs by AGO2-WT and AGO2 variants. Significance was defined by FDR < 0.05 and |log2 fold change|> 1. miRNAs depleted in the variants (relative to AGO2-WT) are shown in blue, enriched miRNAs are shown in orange. Triangles indicate miRNAs detected exclusively in either AGO2-WT or a depicted *AGO2* variant, while circles indicate miRNAs detected in both

In differential enrichment analyses the p.(Asp619Asn) and p.(Phe182del) variants displayed the most pronounced alterations in miRNA association, with 33 and 22 significantly altered miRNAs, respectively (Fig. 5; Additional File 2: Figs. S8, S9). These data are reminiscent of our recent analysis of the p.(Leu192Pro) variant in murine neurons, where we also observed an altered composition of miRNAs bound to the mutant AGO2 protein [11]. The p.(Gly604Arg) and p.(Asn359His) variants exhibited only four significantly differentially bound miRNAs (Figs. 5; S9). A subset of miRNAs exhibited overlapping differential binding across multiple *AGO2* variants, indicating partially shared perturbations in AGO2–miRNA interaction profiles (Additional File 2: Fig. S10A). For instance, miR-1910-5p showed markedly reduced association with the p.(Asp619Asn), p.(Phe182del), and p.(Gly604Arg) variants, while miR-375-3p displayed decreased binding to p.(Phe182del), p.(Gly604Arg), and p.(Asn359His) variants. Similarly, miR-345-5p, miR-589-5p, and miR-769-5p demonstrated pronounced reductions in association with the p.(Asp619Asn) and p.(Phe182del) variants (Additional File 2: Fig. S10B). The overall pattern reveals variant specific effects on miRNA association, with p.(Asp619Asn) and p.(Phe182del) variants showing the strongest deviations from WT, while p.(Gly604Arg) and p.(Asn359His) variants exert only minimal effects.

**Fig. 6:**
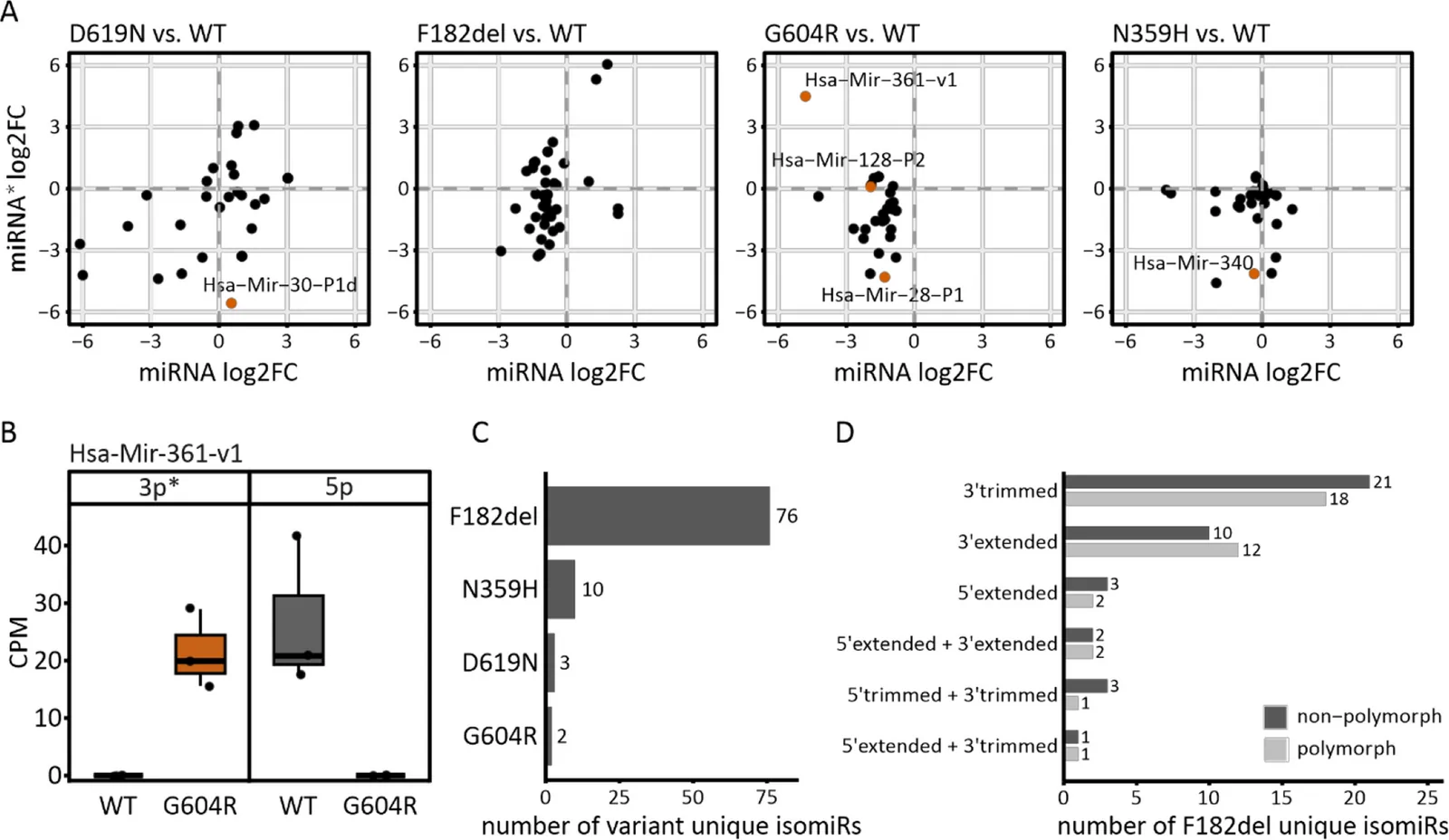
Effect of *AGO2* variants on strand selectivity and isomiR binding. **A** Scatterplot of log2 fold changes (FC) of miRNA guide strands comparing AGO2 variants and AGO2-WT on the x-axis and for the log2FC of the miRNA passenger strand on the y-axis. Significance was assessed at FDR < 0.05 from a linear model fitted to the log2(passenger/guide) CPM ratio. miRNAs with a significant change in the passenger-to-guide ratio in AGO2 variants vs. AGO2-WT are highlighted in orange. **B** Complete strand switch of hsa-miR-361-v1 bound to p.(Gly604Arg)-AGO2 in comparison to AGO2-WT. **C** Number of isomiRs exclusively bound to AGO2 variants (absent in AGO2-WT). **D** Distribution of isomiR categories summarizing isomiRs exclusively detected in the p.(Phe182del) variant but not in AGO2-WT. isomiRs with sequence substitution are shown in light grey (polymorph), while isomiRs without sequence substitution are shown in dark grey (non-polymorph)

To examine whether *AGO2* variants influence strand selection during miRNA loading, we compared the relative association of guide (miR) and passenger (miR*) strands across *AGO2* variants and WT-AGO2 using MA plots. For each variant, log₂ fold changes (variant vs. WT) were plotted against log₁₀-transformed abundance values for both guide and passenger strands. Across all variants, the majority of miRNAs exhibited comparable guide-to-passenger strand ratios relative to WT-AGO2 (Fig. 6A and Additional File 2: Fig. S11), indicating that the overall strand selection mechanism remains largely preserved. However, five specific miRNAs showed significant deviations in strand bias. A complete switch in strand preference was observed for miR-361-v1 in the p.(Gly604Arg) variant (Fig. 6B). Furthermore, several individual miRNAs exhibited altered guide-to-passenger (miR/miR*) ratios in the p.(Asp619Asn), p.(Gly604Arg), and p.(Asn359His) variants, indicating subtle yet measurable effects on strand selection fidelity (Additional File 2: Fig. S12). Collectively, these findings suggest that while AGO2 strand selection is largely conserved across variants, some can perturb the fine balance between guide and passenger strand incorporation of isolated miRNA´s, potentially affecting miRNA-mediated gene silencing fidelity and target specificity [42].

Finally, we assessed whether *AGO2* variants influence the binding of isomiRs. IsomiRs are miRNA isoforms generated by 5′ or 3′ end trimming or extension, sequence variation (polymorphic), or by combined length and sequence alterations (mixed isomiRs [43]). We analyzed isomiRs uniquely associated with AGO2 variants in comparison to AGO2-WT. Quantification revealed that the p.(Phe182del) variant bound the highest number of unique isomiRs (76 isomiRs), followed by p.(Asn359His) (10 isomiRs), whereas p.(Gly604Arg) and p.(Asp619Asn) variants displayed markedly fewer specific isomiRs (Fig. 6C). In combination with our recent findings [11], this further indicates that *AGO2* variants located in the vicinity of helix-7 or the hinge region that enables helix-7 movements can result in an increased diversity of bound miRNA isoforms.

Further classification revealed that the vast majority (~ 93.5%) of isomiRs detected exclusively in the p.(Phe182del) variant corresponded to 3′-modified isomiRs, whereas less than 6.5% represented exclusively 5′-edited isomiRs (Fig. 6D and Additional File 3: Table S2). Interestingly, although p.(Asn359His) exhibited substantially fewer altered isomiRs compared to p.(Phe182del), all ten represented 3′-edited isomiRs (Additional File 4: Table S3). Given the localization of p.(Asn359His) within the helix-7 region, which contributes to miRNA 3′-end accommodation and conformational dynamics of AGO2 [44], this selective enrichment pattern is consistent with a biologically meaningful perturbation of AGO2–miRNA interactions. This altered AGO2–miRNA dynamics closely mirrors the pattern previously observed for the p.(Leu192Pro) *AGO2* variant in murine neurons [11]. We hypothesize that variants affecting the helix-7 region or its associated hinge dynamics impair stabilization of the miRNA 3′ end within the AGO2 complex. This would expose the 3′ end to enzymatic attacks by exonucleases and polymerases [45], thereby promoting the accumulation of 3′ isomiRs and perturbing the fidelity of miRNA-mediated post-transcriptional gene regulation.

Besides providing evidence supporting the pathogenicity of the p.(Asn359His) variant, these analyses reveal that LESKRES-associated *AGO2* variants differentially modulate miRNA binding, strand discrimination, and association with isomiRs. Variants located in regions critical for helix-7 dynamics appear to destabilize the miRNA-AGO2 complex, resulting in increased 3′-end modification and diversification of bound miRNAs. This may contribute to disrupted post-transcriptional regulation and to the observed variability in clinical severity. Nevertheless, despite these mechanistically coherent findings, the currently available evidence does not yet fulfil formal ACMG criteria for likely pathogenic classification, and p.(Asn359His) therefore remains classified as a variant of uncertain significance.

## Discussion

We markedly expand the clinical and molecular spectrum of the *AGO2*-related neurodevelopmental disorder LESKRES, by presenting 45 individuals harboring 33 distinct *AGO2* variants, including 30 novel ones. The recurrent clustering of variants in the L1 loop, in helix-7, and in multiple loops within the PIWI domain underscores the presence of structural hotspots essential for the function of AGO2. Additionally, these findings indicate that variants in diverse AGO2 domains can each lead to neurodevelopmental delay, thereby substantially broadening the variant landscape associated with LESKRES.

Our data expand the phenotypic spectrum associated with *AGO2* variants. Across the combined cohort of 70 individuals (from this and previous studies), delayed speech and language development, intellectual disability, and motor delay remain as hallmark features, affecting nearly all individuals. The near-universal presence of speech delay (97%) and intellectual disability (97%) underscores the critical role of AGO2 in neuronal maturation and cognitive development. The range of cognitive impairment further supports variable expressivity, suggesting the influence of genetic background or environmental modifiers. For example, individual 32 (p.Asp619Asn) presented with neonatal respiratory insufficiency but later demonstrated age-appropriate neurodevelopment, illustrating the potential for functional compensation or reversibility of early deficits.

The neurological phenotype of LESKRES extends beyond developmental delay. Muscular hypotonia was observed in the majority of individuals (69%), likely contributing to delayed motor milestones and feeding difficulties. Behavioural abnormalities, including autistic features (58%) and attention deficit hyperactivity disorder (56%), were also prevalent, aligning with a role of AGO2 in neural circuits implicated in behaviour and cognition. Gait abnormalities (52%) and brain MRI anomalies (44%) further underscore the neurological involvement, suggesting impaired connectivity and cerebellar or cortical dysfunction. Seizures and breathing abnormalities were each present in approximately one-third of individuals, reflecting potential dysfunction in neural networks governing excitability and autonomic control. The co-occurrence of seizures and respiratory issues in some cases indicates that AGO2 dysfunction disrupts both cortical and brainstem development.

Beyond the nervous system, skeletal anomalies (40%), including clinodactyly, craniofacial malformations, and growth abnormalities, were frequent. Additionally, gastrointestinal disturbances (30%) were common, potentially secondary to autonomic dysregulation. Short stature (25%) and congenital heart defects (18%) were also observed, as were urogenital anomalies in approximately one-fifth of affected males. Craniofacial dysmorphism was a recurring but non-specific feature, with epicanthic folds, thin upper lip, and open-mouth appearance among the most prevalent findings. Both microcephaly (four individuals) and progressive macrocephaly (eight individuals) occurred, suggesting that AGO2 dysfunction may perturb developmental pathways regulating neuronal proliferation and growth in divergent directions.

Previously we had proposed that certain clinical features might be restricted to specific *AGO2* variants, implying variant-specific pathomechanisms. In the present cohort, however, this assumption does not appear to hold. Neonatal respiratory failure, manifesting as neonatal apnea, respiratory insufficiency, or pulmonary hypertension, was observed in eleven affected individuals harboring diverse *AGO2* variants (Additional File 1: Table S1). This observation indicates that early respiratory involvement is not confined to the p.(Pro192Leu) variant, as previously postulated, but rather represents a recurrent manifestation across multiple alleles, suggesting a shared pathogenic mechanism affecting early developmental or autonomic control pathways. Similarly, clinodactyly of the fifth finger, initially thought to be specific to p.(Cys751Tyr), was identified in five additional individuals: two harboring p.(Gly604Arg), one harboring p.(Pro602His), one harboring p.(Pro602Arg) and one (individual 42) harboring p.(Arg795Cys). These findings further support the notion of overlapping phenotypic spectra among distinct *AGO2* variants. Moreover, we identified additional features, including hydronephrosis in individual 17 p.(Ile365Val) and precocious puberty in individual 20 p.(Pro415Leu), highlighting a broader systemic impact than previously appreciated. Collectively, these observations suggest that, although certain genotype–phenotype correlations may exist, the clinical expression of AGO2-related neurodevelopmental disorder is more variable and interconnected than initially anticipated, likely reflecting convergent downstream effects of distinct molecular perturbations within the same functional pathway. The variability in clinical severity and phenotypic presentation among individuals carrying identical variants implies the influence of additional genetic or epigenetic modifiers. For example, clinodactyly of the fifth finger was absent in one individual with p.(Pro602Arg) and in two of four individuals with p.(Gly604Arg). Among the latter, one individual exhibited age-appropriate motor development, and two presented with macrocephaly. Macrocephaly was also observed in two of four affected individuals from the same family harboring the p.(Ser180Phe) variant, further suggesting intrafamilial variability. Additionally, individual 39, the affected father of individual 38, appeared to have a milder clinical course compared to his son; however, due to the absence of clinical data from his early childhood, a direct comparison is impossible.

The clinical variability may reflect distinct mechanistic consequences of individual variants on AGO2 function, including altered protein–protein interactions, impaired subcellular localization, or dysregulated miRNA binding kinetics. Indeed, distinct *AGO2* variants affect AGO2 function through diverse perturbations of its regulatory dynamics. In comparison to our initial studies, where all tested *AGO2* variants impaired shRNA-mediated silencing, this was observed here only for p.(Arg714Trp) and p.(Asn729His). These findings are in agreement with the fact that siRNA-mediated gene silencing plays a relatively limited physiological role in humans. Unlike plants and some invertebrates, humans lack RNA-dependent RNA polymerases and therefore do not generate endogenous small interfering RNAs (siRNAs) [46].

Three variants, p.(Arg255Ser), p.(Asn359His), and p.(Arg761Trp), currently remain classified as variants of uncertain significance according to strict ACMG criteria. Nevertheless, several lines of evidence support their likely contribution to disease. The p.(Arg255Ser) variant affects a highly conserved residue for which a pathogenic variant involving the corresponding amino acid in *AGO1*, p.(Arg253His), has previously been reported in Neurodevelopmental disorder with language delay and behavioral abnormalities, with or without seizures (NEDLBAS, [10]), further supporting functional importance of this Argonaute residue. Although the functional consequences of p.(Asn359His) were quantitatively more subtle than for other LESKRES-associated variants, this variant localizes to the helix-7 region and selectively enriched 3′-edited isomiRs, a pattern highly reminiscent of p.(Leu192Pro) and p.(Phe182del). Finally, p.(Arg761Trp) is located within the PIWI domain in close proximity to the AGO2 regulatory phosphorylation cluster and structurally dynamic surfaces involved in target RNA release and cofactor interactions. Given the critical role of PIWI-domain conformational dynamics in AGO2-mediated gene silencing, perturbation of this region is likely to affect AGO2 regulatory cycling and RISC function. Collectively, these observations support the biological relevance of these variants, although the currently available evidence remains insufficient for formal likely pathogenic classification.

Mechanistically, our interaction studies provide insight into how specific variants influence AGO2 molecular behavior. The p.(Asp619Asn) variant displayed reduced affinity for GW182, implicating this residue in scaffolding interactions that underlie mRNA degradation and RISC recycling. Asp619 is located at the entrance of one of the three tryptophan binding sites on the surface of the PIWI domain (Additional File 2: Fig. S13). Binding to GW182 is reduced but not completely lost. It appears likely that one of the tryptophan pockets in the PIWI domain is rendered non-functional in this variant, thereby preventing a supporting role of the expressed AGO2 protein in establishing larger clusters of P-body associated proteins. As a consequence, the mutant AGO2 protein failed to localize to P-bodies, and even suppressed P-body formation in HEK293T cells. It is unclear why we did not observe this effect in hippocampal neurons, but it should be noted that we avoid the use of the strong CMV-promoter in neurons. Therefore, the negative effect of the p.(Asp619Asn) protein on P-body formation may have been not as strong as in HEK293T cells.

Reduced phosphorylation was observed for p.(Arg506Gln), p.(Glu531Gln), p.(Gly604Arg) and p.(Asp619Asn) variants. Impaired phosphorylation may delay target release and prolong stability of the complex of AGO2–miRNA with its mRNA targets [35, 36]. This supports a model in which multiple structural perturbations can converge on a shared pathogenic mechanism: altered turnover of AGO2-miRNA complexes on their mRNA targets.

Our data reveal that *AGO2* variants differentially affect miRNA association, strand selection, and isomiR composition. p.(Phe182del) and p.(Asp619Asn) variants caused the most extensive alterations in miRNA binding, whereas p.(Gly604Arg) and p.(Asn359His) exhibited quantitatively more modest alterations. However, interpretation of these more subtle changes remains challenging, particularly in the absence of suitable benign control variants located within the same functional regions, namely helix-7 for p.(Asn359His) and loop 1 of the PIWI domain for p.(Gly604Arg). Importantly, overlapping patterns of altered miRNA association across multiple variants indicate partially shared defects in miRNA handling. Whereas the core mechanism of guide versus passenger strand incorporation remained largely preserved across variants, we observed a complete reversal of strand preference for miR-361-v1 in the case of p.(Gly604Arg). Such imbalance may distort miRNA-target recognition and contribute to cumulative post-transcriptional dysregulation. IsomiR profiling uncovered variant-specific enrichment of 3′-modified miRNAs, particularly for p.(Phe182del). Notably, although the p.(Asn359His) variant exhibited substantially fewer altered isomiRs overall, all significantly enriched ones represented 3′-edited isomiRs, suggesting a selective disturbance of miRNA 3′-end handling. Given the localization of Asn359 within the helix-7 region, which regulates AGO2 conformational dynamics and miRNA accommodation, this finding further supports the functional importance of helix-7-associated LESKRES variants. The predominance of 3′-end modifications suggests a failure to anchor the 3’ end of miRNA within its binding pocket in AGO2. This may lead to increased susceptibility to exonucleolytic or uridylation events. The strong resemblance between effects of the p.(Phe182del) variant and the previously reported alterations in p.(Leu192Pro)-associated isomiR profiles [11] supports a shared mechanism. These findings raise the possibility that sequence variation within miRNAs themselves may contribute to interfamilial variability and clinical severity among individuals with identical *AGO2* variants. Investigating this hypothesis will require a multi-omics approach integrating whole-genome and miRNA-sequencing of multiple individuals harbouring the identical variant. Future studies integrating genome and miRNA sequencing across individuals harbouring identical *AGO2* variants will be required to address this possibility.

Collectively, these mechanistic findings also have important implications for interpretation of functional evidence in *AGO2*-associated disease. AGO2 is a highly specialized and evolutionarily constrained component of the RNA-induced silencing complex (RISC), mediating multiple tightly interconnected processes including miRNA loading, strand selection, target recognition, GW182 recruitment, phosphorylation-dependent target release, P-body dynamics, and isomiR handling. Consequently, distinct pathogenic variants may perturb different aspects of AGO2 biology without necessarily producing identical experimental phenotypes. Moreover, *AGO2* currently lacks a sufficiently large set of well-established benign missense variants suitable for formal calibration of functional assays across individual structural domains. Therefore, in accordance with current ClinGen recommendations for PS3/BS3 interpretation [19], we evaluated functional evidence by considering biological relevance, assay specificity, reproducibility, use of appropriate positive and negative controls, and concordance across independent AGO2-specific functional assays rather than relying on a single assay type alone (Additional File 1: Table S1). Variants demonstrating consistent abnormalities across multiple independent AGO2 functional readouts, such as p.(Asp619Asn) and p.(Arg714Trp), were therefore interpreted as providing stronger functional evidence than variants exhibiting more restricted or domain-specific abnormalities. In contrast, variants with subtler phenotypes, including selective phosphorylation defects, altered strand selectivity, or isomiR-specific perturbations, were interpreted more conservatively. Taken together, these findings illustrate that LESKRES-associated variants can differentially impair distinct layers of AGO2-mediated post-transcriptional regulation while converging on a shared neurodevelopmental phenotype.

## Conclusions

Our results delineate a multifaceted model of AGO2 dysfunction in LESKRES: pathogenic variants disrupt distinct yet interconnected processes: P-body association, phosphorylation-dependent turnover, miRNA binding and isomiR generation. The expanded cohort establishes LESKRES as a neurodevelopmental disorder with a core triad of speech delay, intellectual disability and motor impairment, accompanied by variable neurological, behavioural, visual and other systemic features. By integrating deep phenotyping with complementary functional analyses, we demonstrate how distinct *AGO2* variants perturb different layers of post-transcriptional gene regulation while converging on a shared neurodevelopmental phenotype. Collectively, these findings highlight the critical importance of AGO2 conformational dynamics and precise AGO2–miRNA interactions for human neuronal development.

## Supplementary Information

Additional file 1: Table S1. This Table contains clinical features of all patients reported in this manuscript.

Additional file 2: Figure S1—S16. All supplementary Figures; and uncropped blots for Figs. 2 and 3 of the main manuscript.

Additional file 3: Table S2. IsomiRs associated with the F182del variant. Table S3. IsomiRs associated with the N359H variant.

## Data Availability

The raw RNA sequence data have been deposited in the gene expression omnibus database (https://www.ncbi.nlm.nih.gov/geo/) under accession number GSE311030. The newly identified AGO2 variants have been deposited to the Leiden Open Variation Database (LOVD) [47 with the variant numbers #0001080193—#0001080237 https://databases.lovd.nl/shared/variants/0001080193 [48] https://databases.lovd.nl/shared/variants/0001080197 [49] https://databases.lovd.nl/shared/variants/0001080199 [50] https://databases.lovd.nl/shared/variants/0001080200 [51] https://databases.lovd.nl/shared/variants/0001080201 [52] https://databases.lovd.nl/shared/variants/0001080202 [53] https://databases.lovd.nl/shared/variants/0001080203 [54] https://databases.lovd.nl/shared/variants/0001080204 [55] https://databases.lovd.nl/shared/variants/0001080205 [56] https://databases.lovd.nl/shared/variants/0001080206 [57] https://databases.lovd.nl/shared/variants/0001080207 [58] https://databases.lovd.nl/shared/variants/0001080208 [59] https://databases.lovd.nl/shared/variants/0001080210 [60] https://databases.lovd.nl/shared/variants/0001080211 [61] https://databases.lovd.nl/shared/variants/0001080212 [62] https://databases.lovd.nl/shared/variants/0001080213 [63] https://databases.lovd.nl/shared/variants/0001080214 [64] https://databases.lovd.nl/shared/variants/0001080215 [65] https://databases.lovd.nl/shared/variants/0001080216 [66] https://databases.lovd.nl/shared/variants/0001080217 [67] https://databases.lovd.nl/shared/variants/0001080218 [68] https://databases.lovd.nl/shared/variants/0001080223 [69] https://databases.lovd.nl/shared/variants/0001080224 [70] https://databases.lovd.nl/shared/variants/0001080226 [71] https://databases.lovd.nl/shared/variants/0001080227 [72] https://databases.lovd.nl/shared/variants/0001080228 [73] https://databases.lovd.nl/shared/variants/0001080229 [74] https://databases.lovd.nl/shared/variants/0001080231 [75] https://databases.lovd.nl/shared/variants/0001080232 [76] https://databases.lovd.nl/shared/variants/0001080234 [77] https://databases.lovd.nl/shared/variants/0001080235 [78] https://databases.lovd.nl/shared/variants/0001080236 [79] https://databases.lovd.nl/shared/variants/0001080237 [80] All other data supporting the findings of this study are available within the paper and its Supplementary material. Materials (e.g. plasmids) are available from the corresponding authors upon request.
