# Supplementary material for: The clinical and molecular spectrum of AGO2-associated Lessel-Kreienkamp neurodevelopmental syndrome
Source: Genome Med. 2026 Aug 22;18:125. doi: 10.1186/s13073-026-01745-4 (PMC13501643; doi:10.1186/s13073-026-01745-4)
Supplement: Supplementary file 1 [file 13073_2026_1745_MOESM2_ESM.pdf]

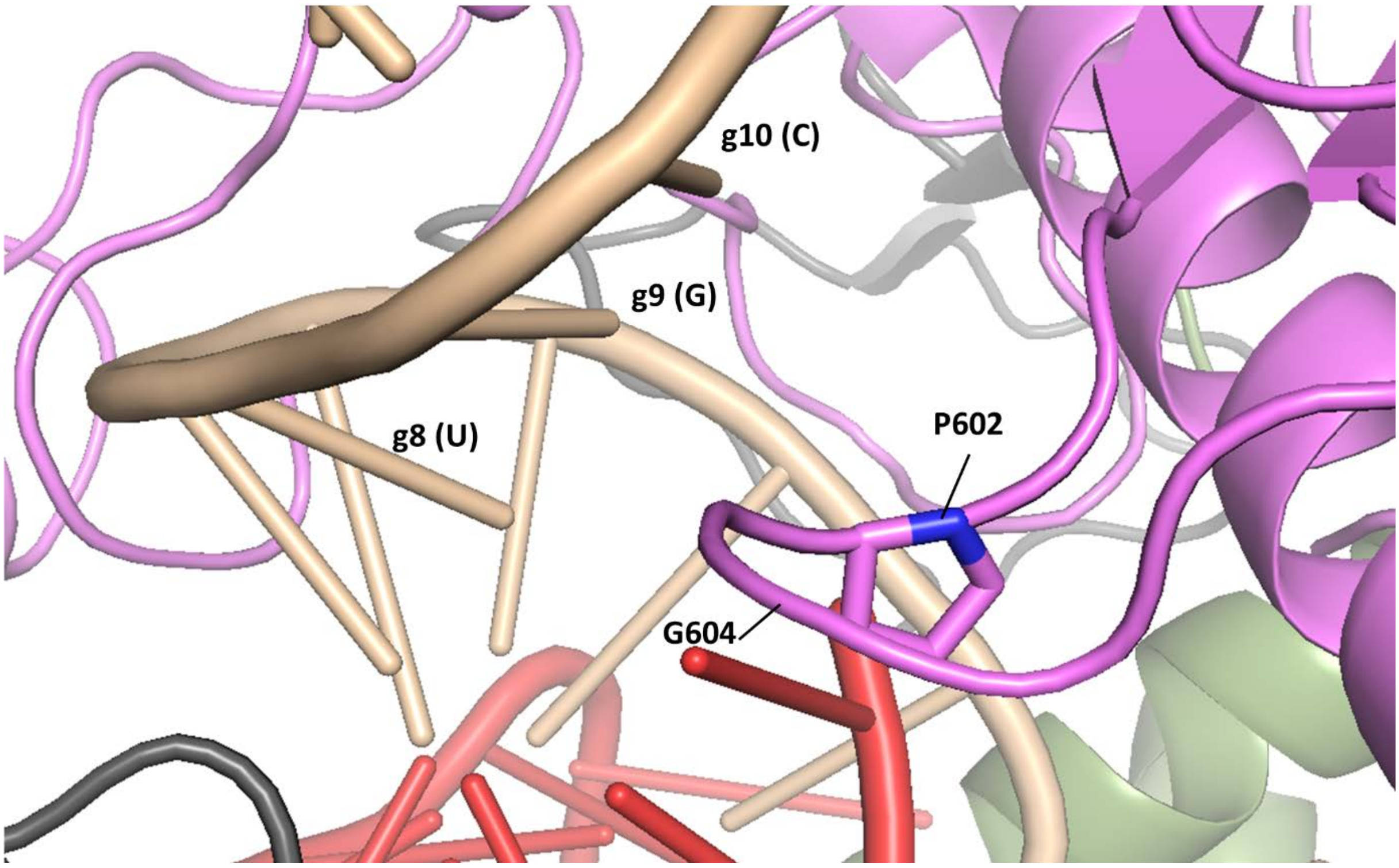

Fig. S1: Detail of the structure of human AGO2 in a complex with guide and target RNAs. Loop 1 of the PIWI domain (violet), which includes P602 and G604, is shown in close contact with base #9 of the guide RNA (wheat; g9, which is Guanine). Bases of guide and target RNAs are shown as sticks only for clarity.

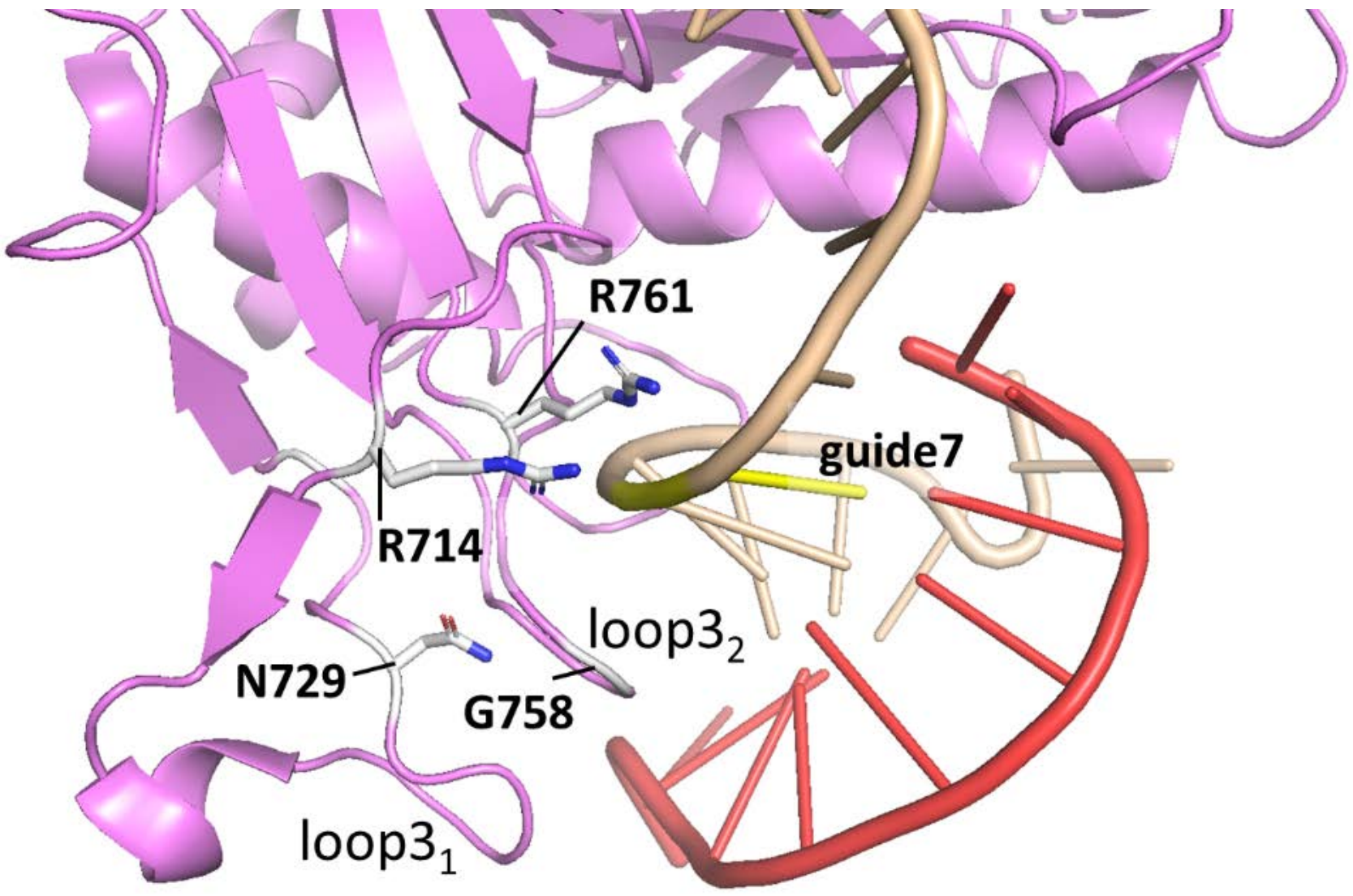

Fig. S2: The duplex of guide RNA (wheat/yellow) and target mRNA (red) is shown. The g7 nucleotide is highlighted in yellow. The PIWI domain of AGO2 is shown in violet; residues found altered in this study (in variants R714W, N729H, G758E, R761W) are indicated in white, with side chains coloured according to the elements. These residues are positioned on two loops (termed loop 3_1 and loop 3_2 by Nakanishi (1)) making extensive contacts to both RNA molecules. Note that the positively charged side chain of R714 (altered to W in individuals 33 and 34) directly contacts the phosphate group of nucleotide g7; N729 (altered to H in individual 35) stabilizes the position of loop 3_2; and the substitution of small G758 to the large, negatively charged Glu (observed in individual 36) appears to be incompatible with its position close to the target mRNA.

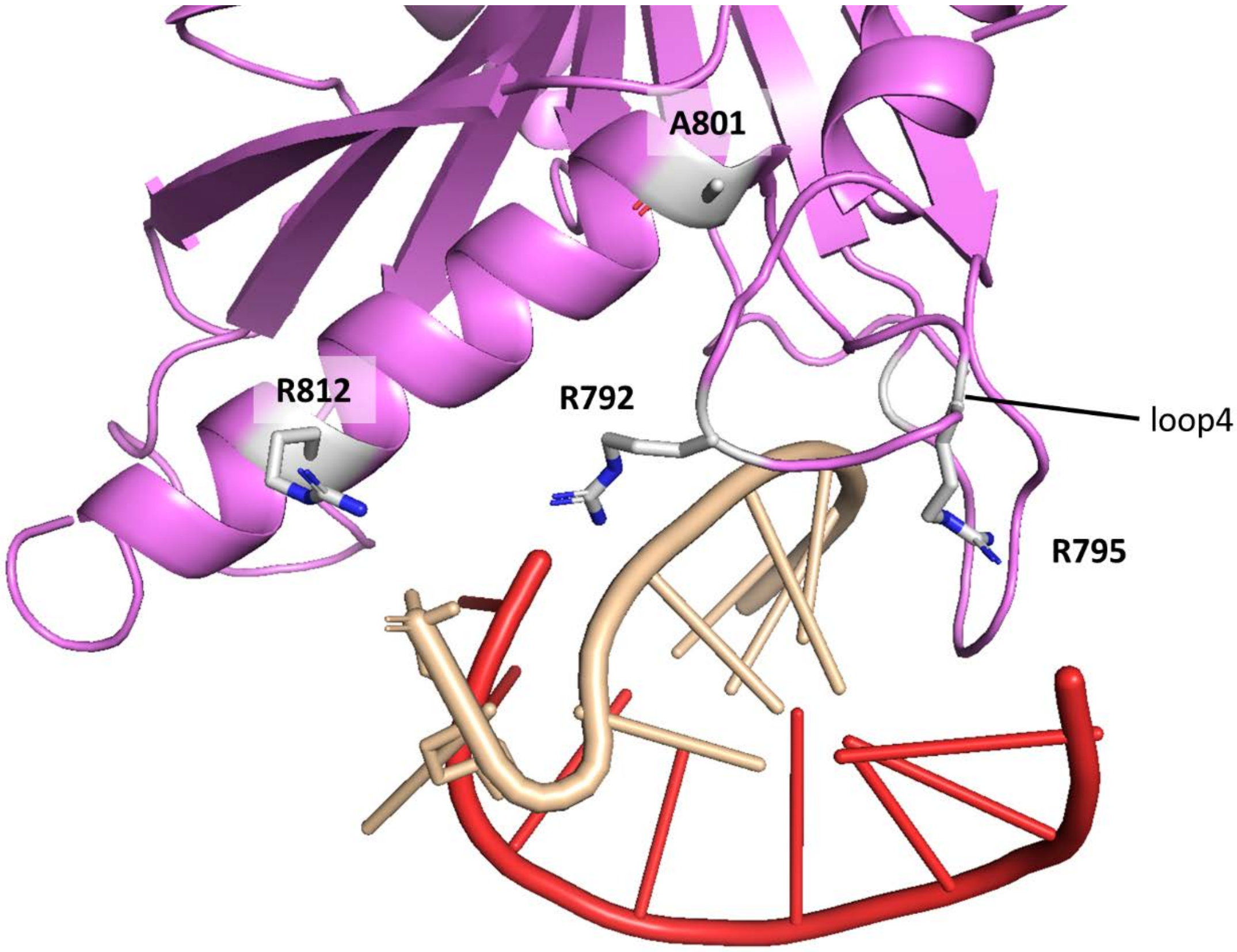

Fig. S3: Another view of the PIWI domain of AGO2 (violet) in complex with the guide RNA (wheat)/target mRNA (red) duplex. Residues R792 (altered to H in individual 40) and R795 (changed to C in patient 41) are located on loop 4 which makes extensive contacts to the guide RNA, thereby helping to shape the seed region of the guide. A801 (mutated to V in individual 42) marks the transition between loop4 and the following α-helix, which carries R812 (changed to Q in patient 43).

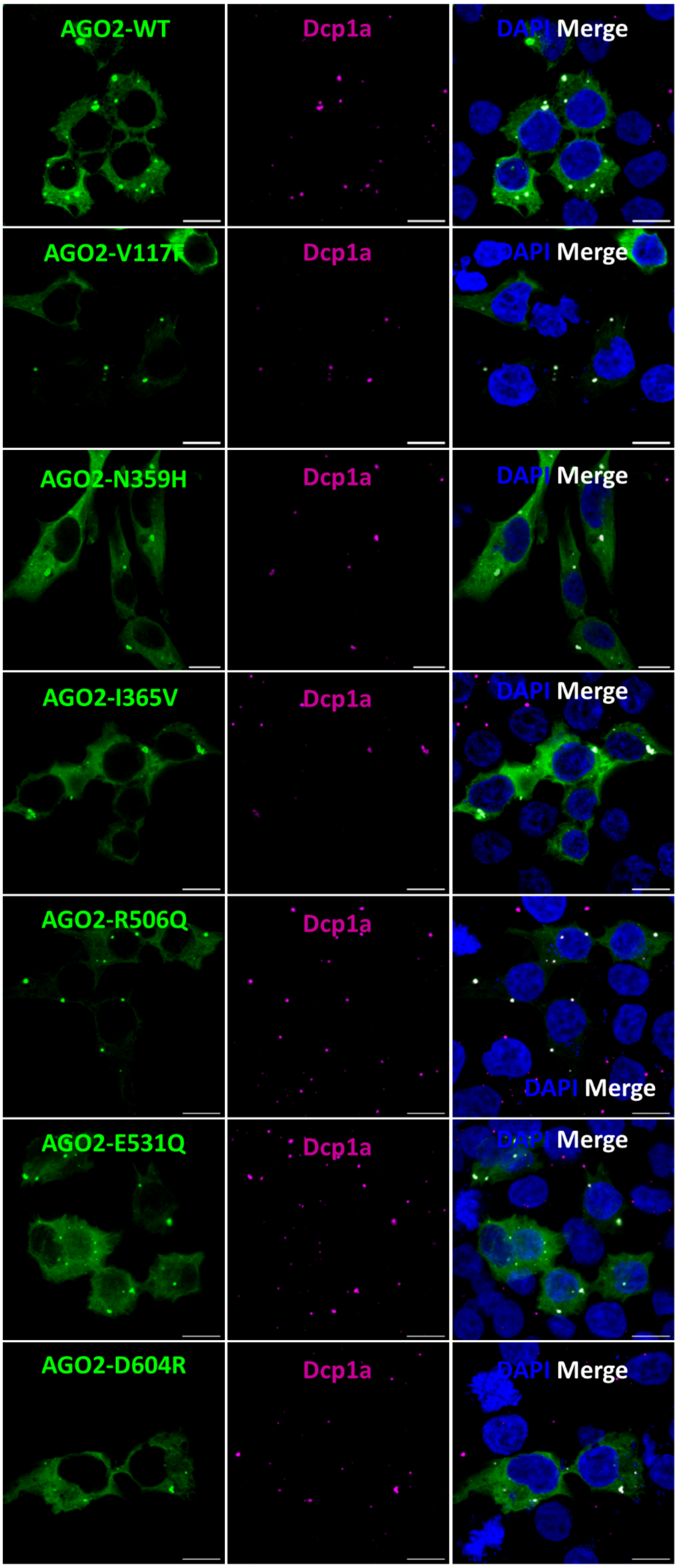
AGO2-WT
Dcp1a
DAPI Merge
AGO2-V117F
Dcp1a
DAPI Merge
AGO2-N359H
Dcp1a
DAPI Merge
AGO2-I365V
Dcp1a
DAPI Merge
AGO2-R506Q
Dcp1a
DAPI Merge
AGO2-E531Q
Dcp1a
DAPI Merge
AGO2-D604R
Dcp1a
DAPI Merge

Fig. S4. Cellular localisation of AGO2-mutants. A. HEK293T cells transfected with plasmids coding for GFP or GFP-tagged AGO2-variants were fixed and stained for Dcp1a (magenta) and nuclear DNA (blue). Cells were analysed by confocal microscopy.

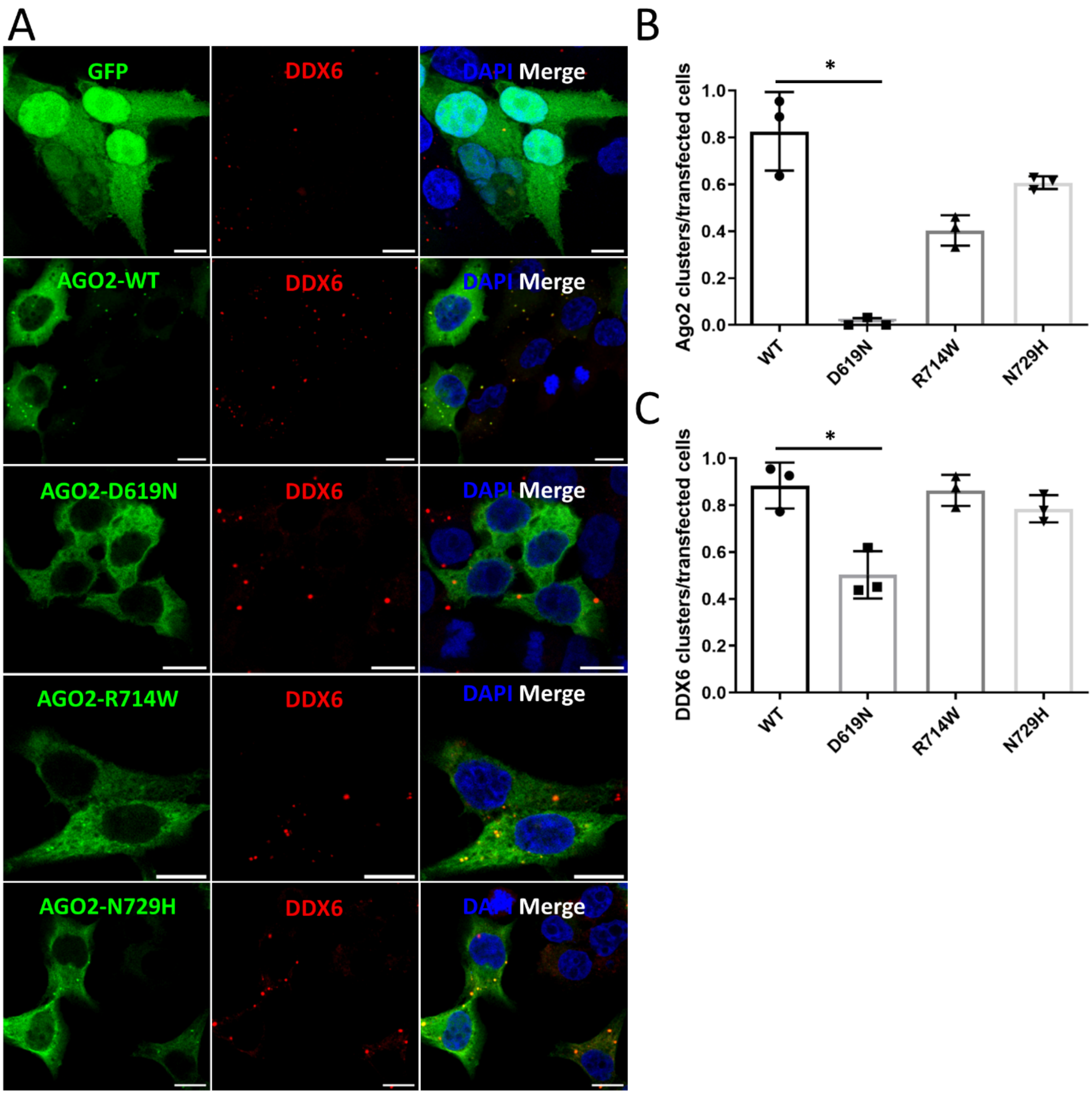

Fig. S5. A. HEK293T cells transfected with plasmids coding for GFP or GFP-tagged AGO2-variants were fixed and stained for DDX6 (magenta) and nuclear DNA (blue). Cells were analysed by confocal microscopy. **B, C.** Quantitative analysis of the number of GFP-AGO2 clusters (B) or DDX6 clusters (C) normalized to the number of transfected cells (C). *, significantly different from WT, $p<0.05$, ANOVA, followed by Dunnett's multiple comparisons test.

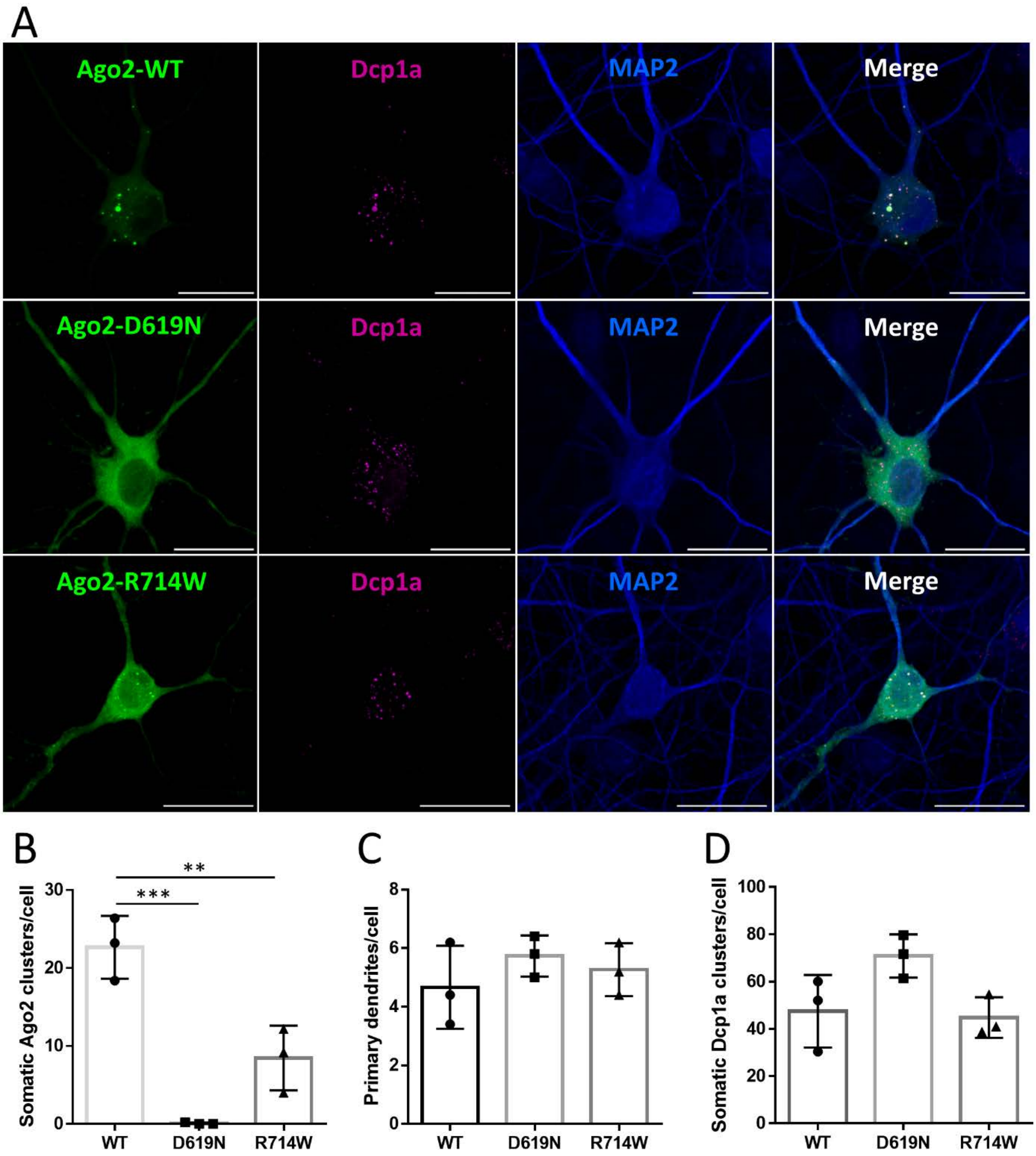

Fig. S6. A. Primary neurons expressing GFP-Ago2 variants were fixed and stained the dendritic marker MAP2, and the P-body marker Dcp1a. **B-D:** Neuronal somata were analysed for the number of Ago2 and Dcp1a clusters per cell, and for the number of primary dendrites per cell. **, ***, significantly different from WT, $p<0.01$, <0.001, respectively; ANOVA, followed by Dunnett's multiple comparisons test (n=3).

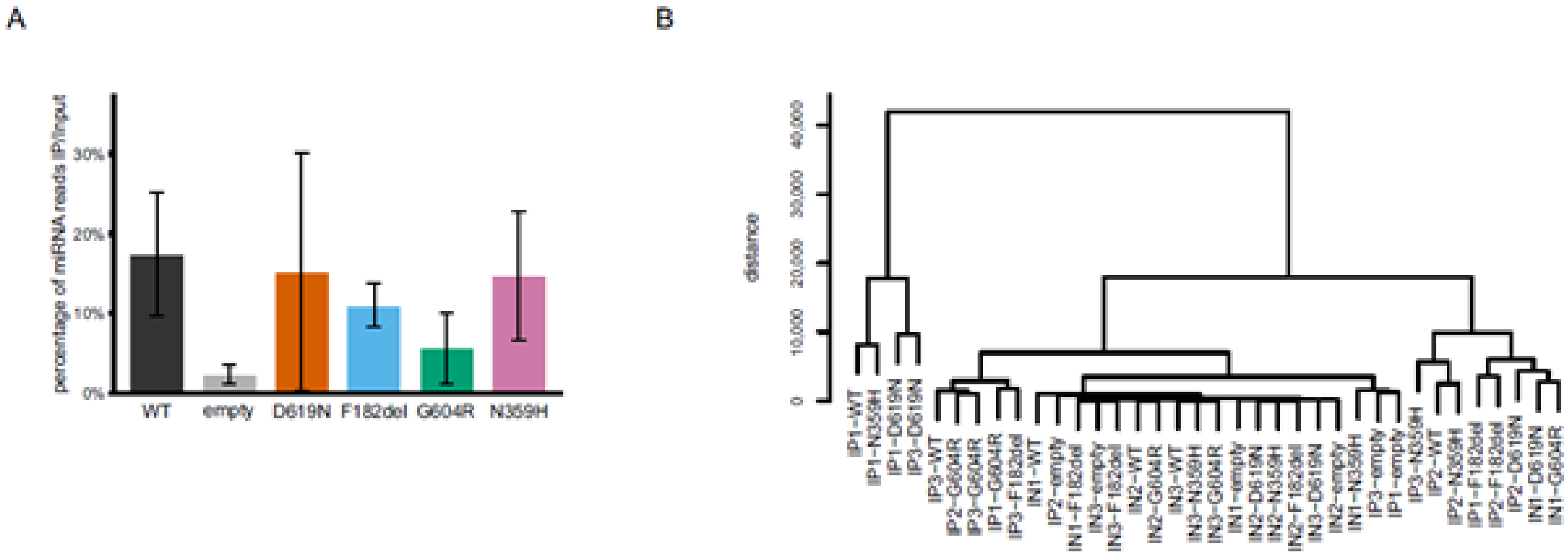

Fig. S7. miRNA enrichment and sample clustering in small RNA-sequencing data. A. Enrichment of miRNA reads in IP versus input samples, quantified as the percentage of miRNA-assigned reads relative to the total read count in the analysis per sample. B. Dendrogram of small RNA-sequencing data based on sRNAtoolbox-processed counts, expressed as reads per million (RPM).

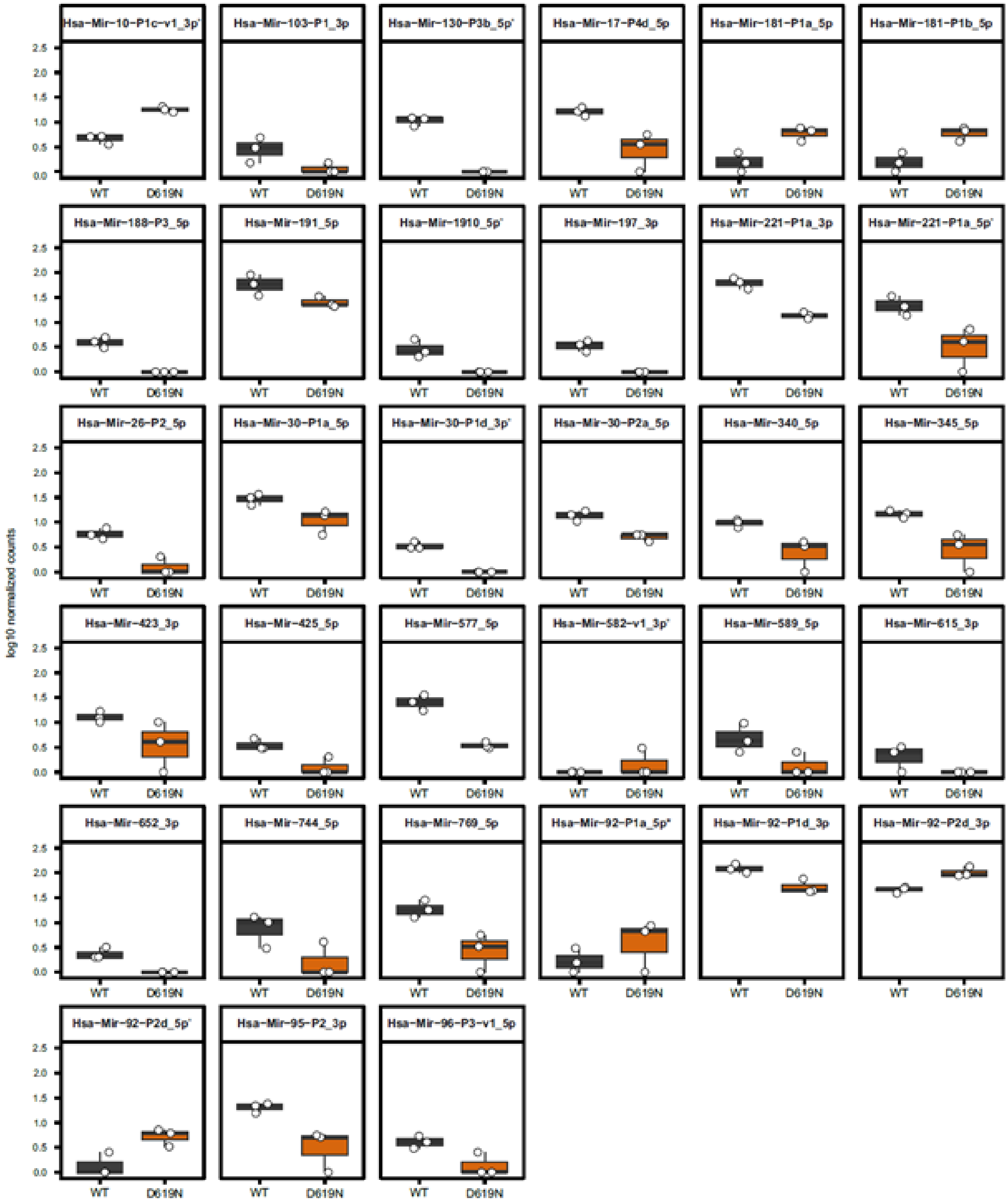

Fig. S8. Differentially bound miRNAs in AGO2-D619N. Boxplots of miRNAs significantly differentially bound between AGO2-WT and AGO2-D619N. Plotted are log10-transformed normalized counts derived from DESeq (as implemented in sRNAde within sRNAtoolbox). Significance was defined as FDR < 0.05 and |log2 fold change| > 1.

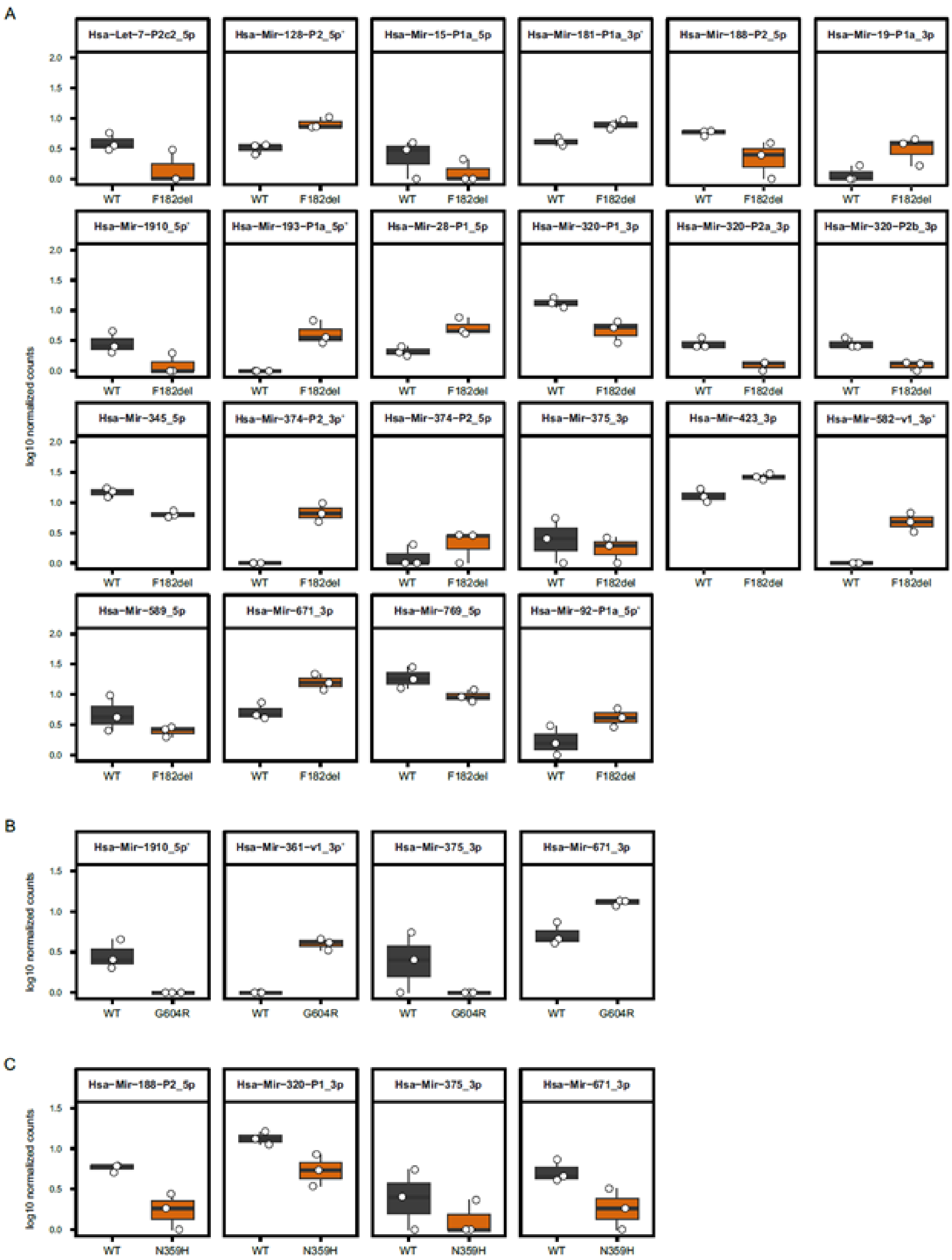

Fig. S9. Differentially bound miRNAs in AGO2 variants F182del, G604R and N359H. Boxplots of miRNAs significantly differentially bound between AGO2-WT and AGO2 variants F182del (A), G604R (B) and N359H (C). Plotted are log10-transformed normalized counts derived from DESeq (as implemented in sRNAde within sRNAtoolbox). Significance was defined as FDR < 0.05 and |log2 fold change| > 1.

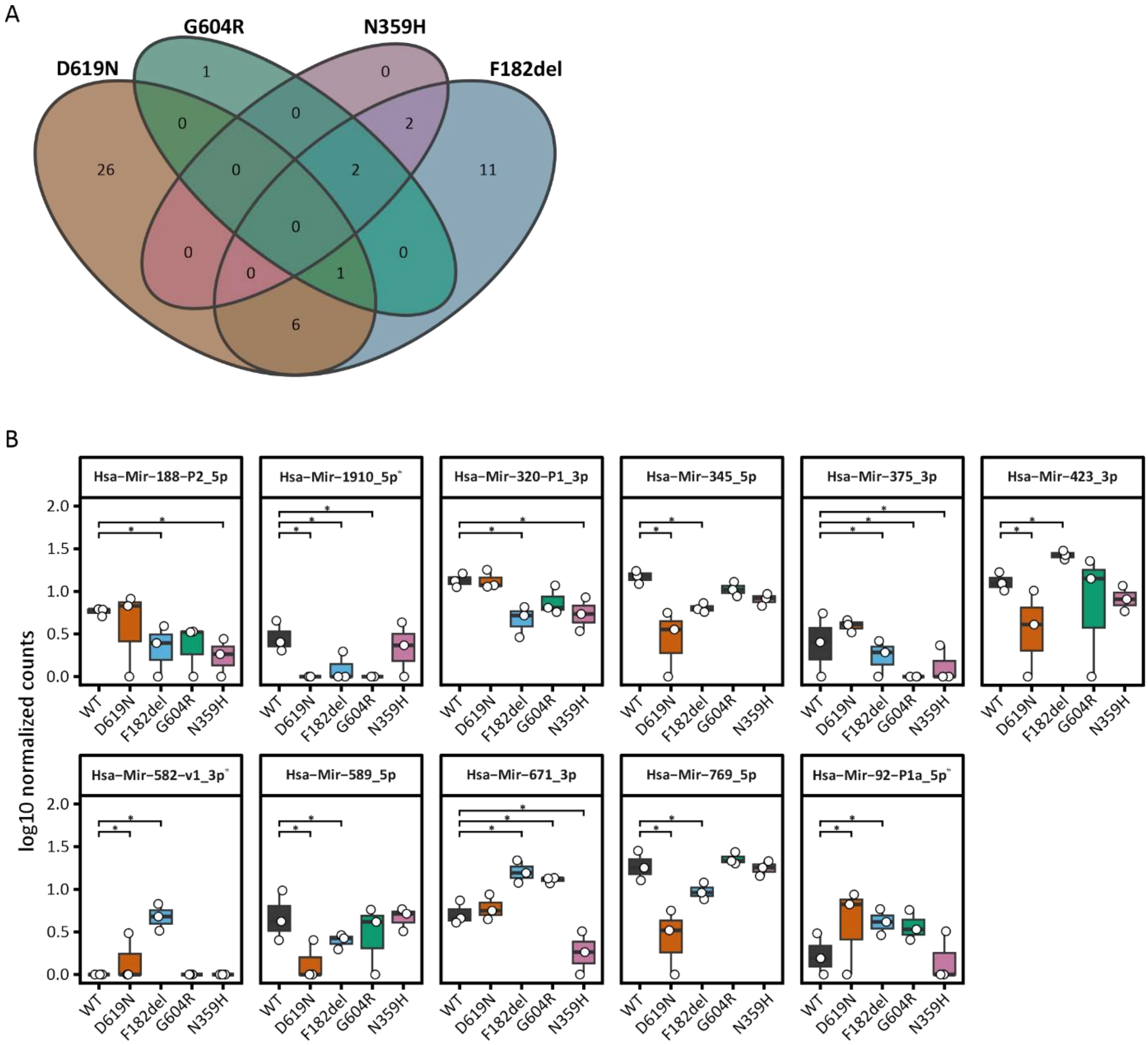

Fig. S10. miRNAs differentially bound across AGO2 variants. A. Venn diagram showing the overlap of miRNAs significantly differentially bound for each AGO2 variant relative to AGO2-WT (FDR < 0.05 and |log2 fold change| > 1). B. Boxplots of miRNAs significantly differentially bound in at least two AGO2 variants versus AGO2-WT. Plotted are log10-transformed normalized counts derived from DESeq (as implemented in sRNAde within sRNAtoolbox). * indicates a significant difference (FDR < 0.05 and |log2 fold change| > 1).

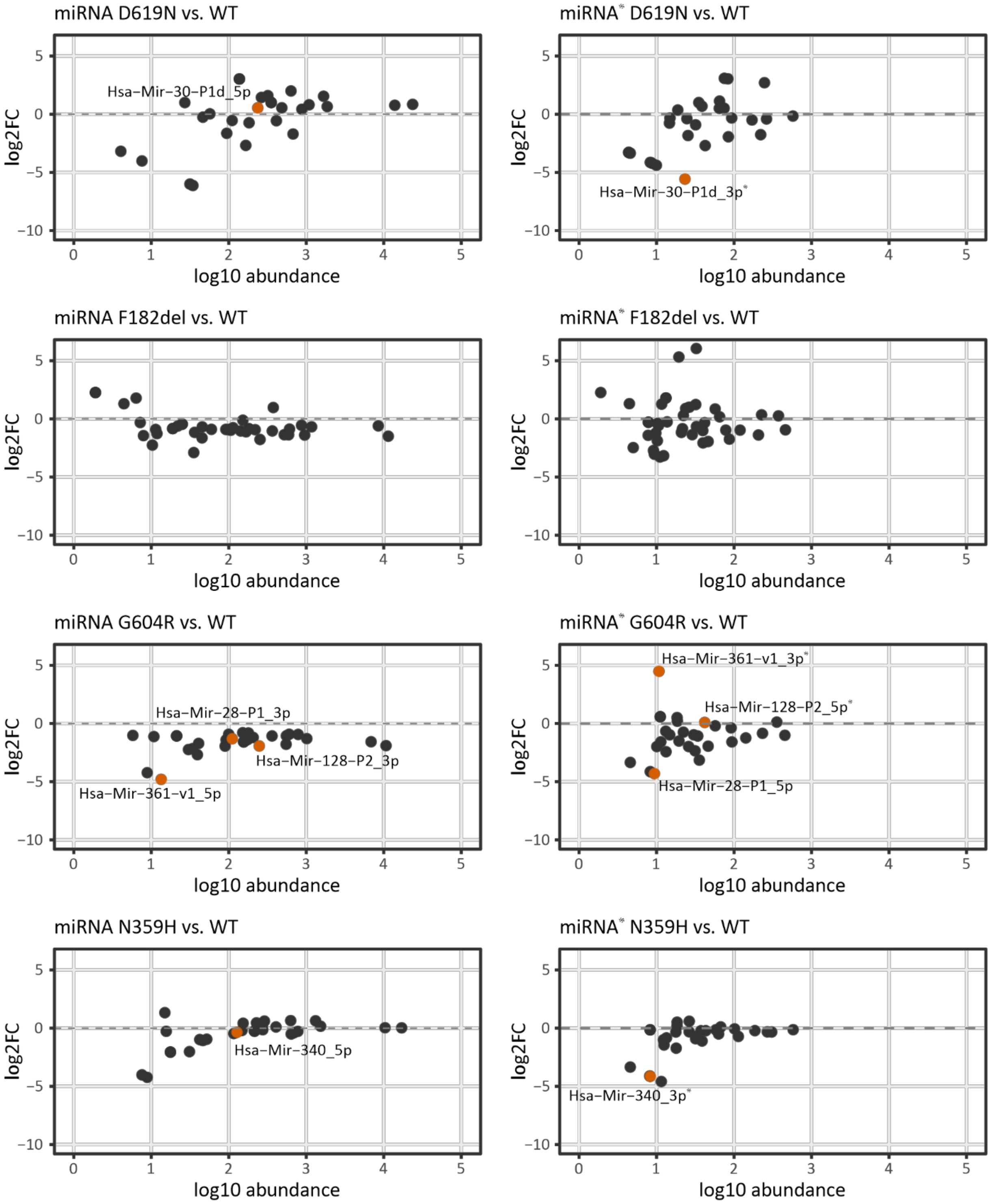

Fig. S11. Strand selectivity of AGO2 variants. MA plots for miRNA guide strands (left) and passenger strands (right) comparing AGO2 variants with AGO2-WT. The x-axis shows log10 abundance, the y-axis shows the log2FC (variant vs. AGO2-WT). Significance was assessed at FDR < 0.05 using a linear model fitted to the log2(passenger/guide) CPM ratio. miRNAs with a significant change in the passenger-to-guide ratio in AGO2 variants vs. AGO2-WT are highlighted in orange.

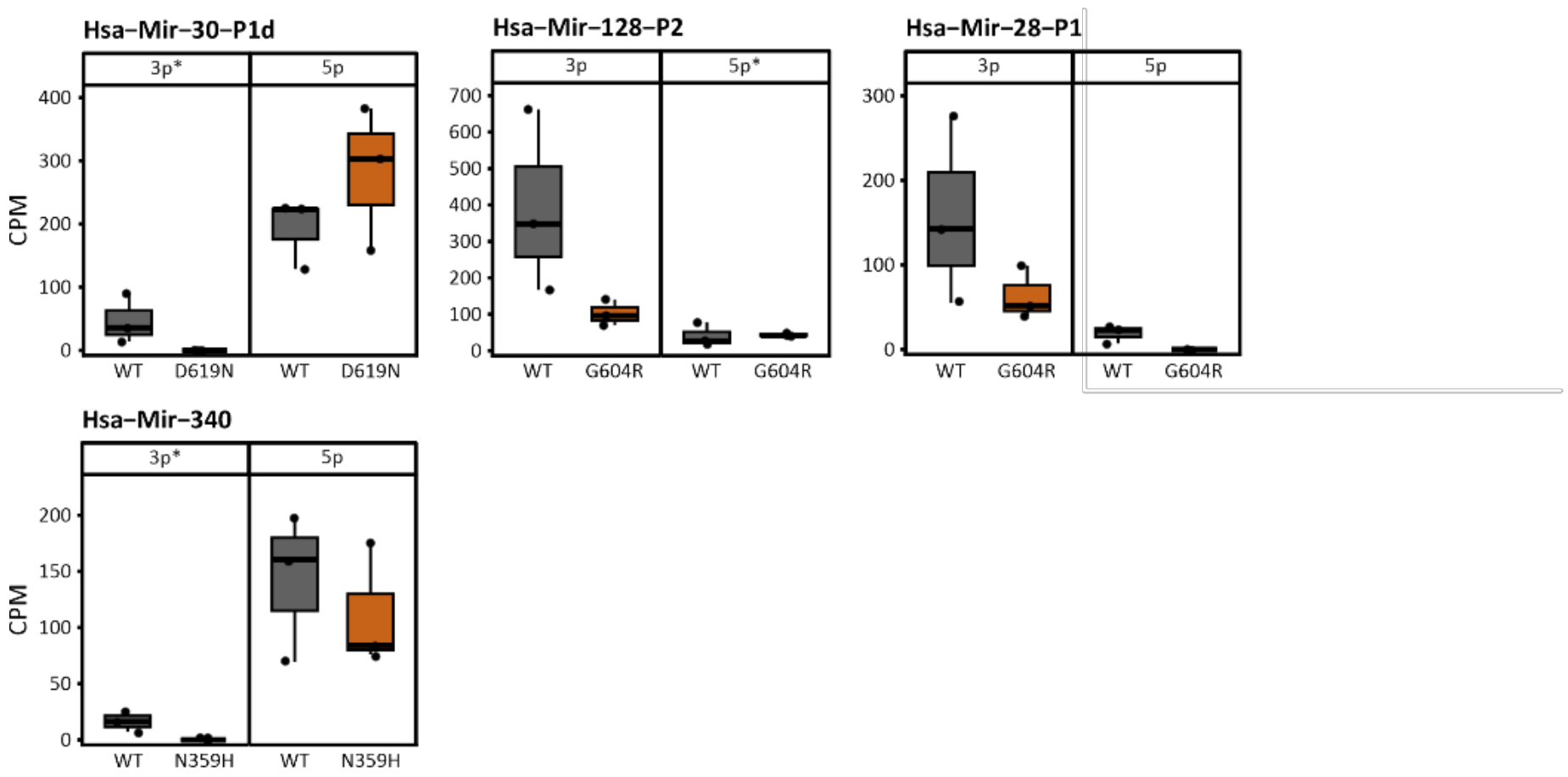

Fig. S12. Abundance of miRNAs with altered strand selectivity. Abundance of miRNA guide and passenger strands that exhibit altered strand selectivity in AGO2 variants relative to AGO2-WT. Values represent AGO2-bound miRNAs quantified as counts per million (CPM). Significance for altered strand selectivity defined from the linear model on log2(passenger/guide) CPM, FDR < 0.05.

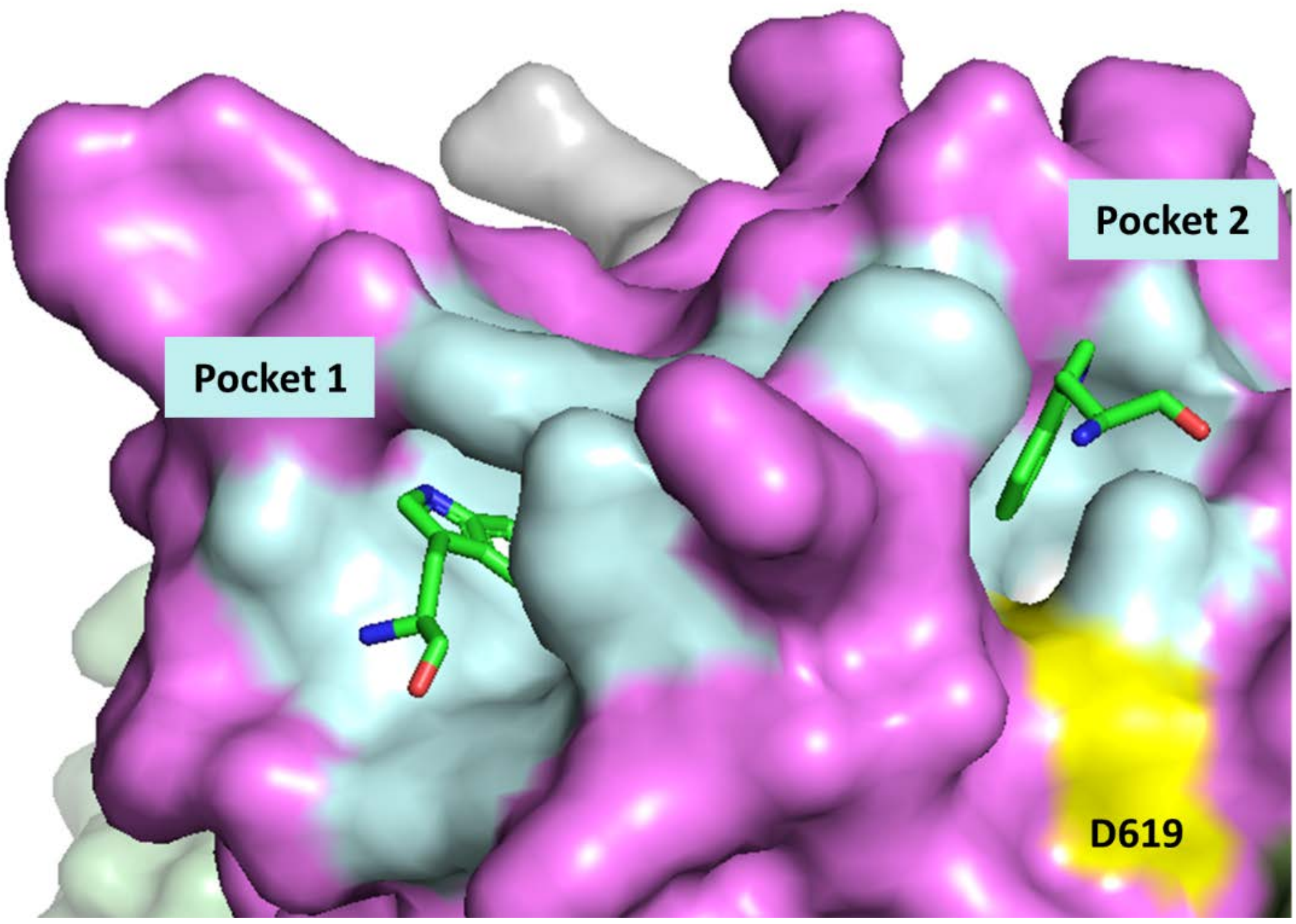

Fig. S13. Surface representation of part of the Ago2 PIWI domain (violet, pale cyan). Parts of the N-domain are also visible. Amino acids lining either of the two pockets for Trp residues of Gw182 are shown in pale cyan. The two tryptophanes are shown as stick models. The position of Asp619 (yellow) at the entrance of pocket 2 is indicated. The model is derived from database entry 4olb.pdb (2).

Uncropped blots Fig. 2a

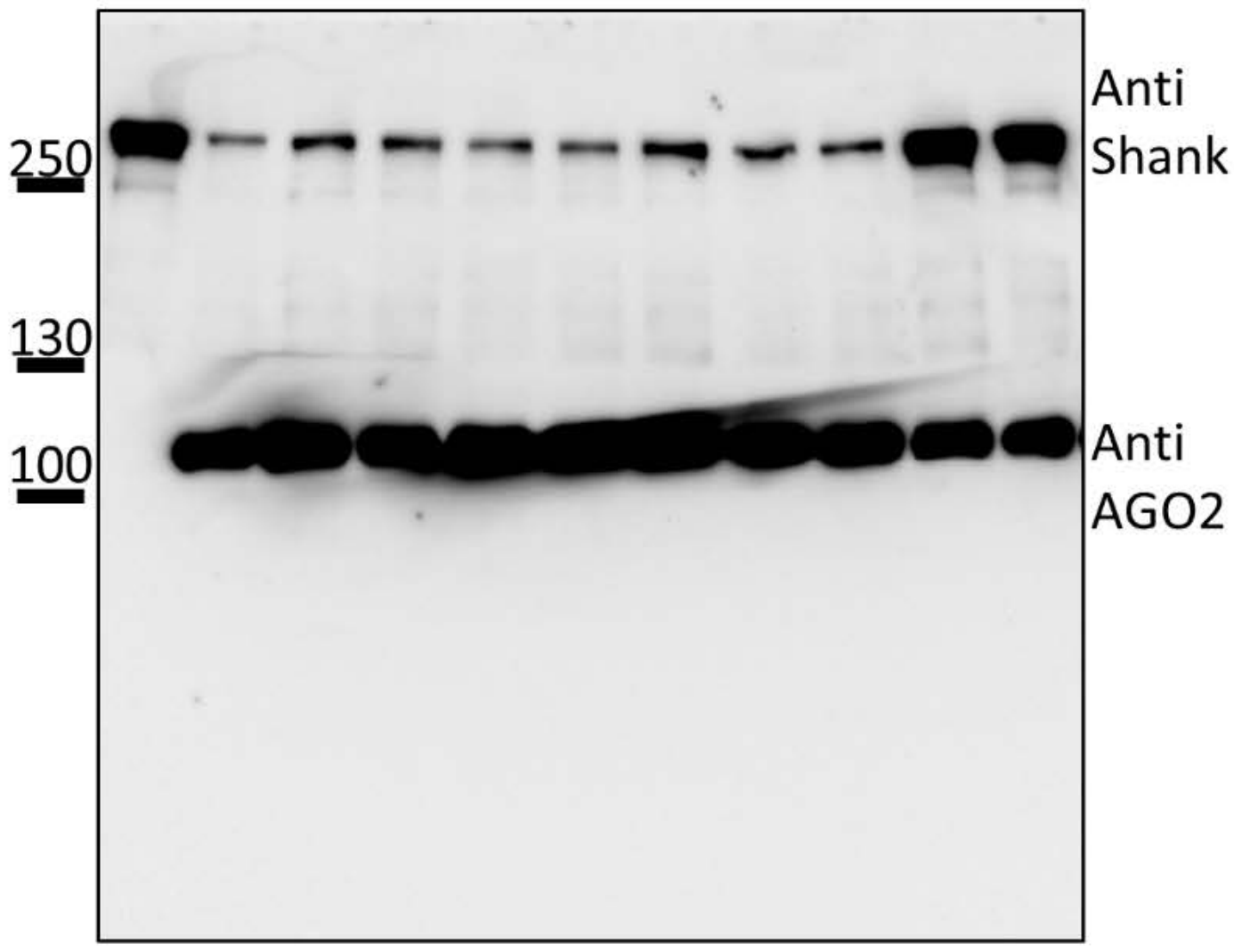

Uncropped blots Fig. 2c

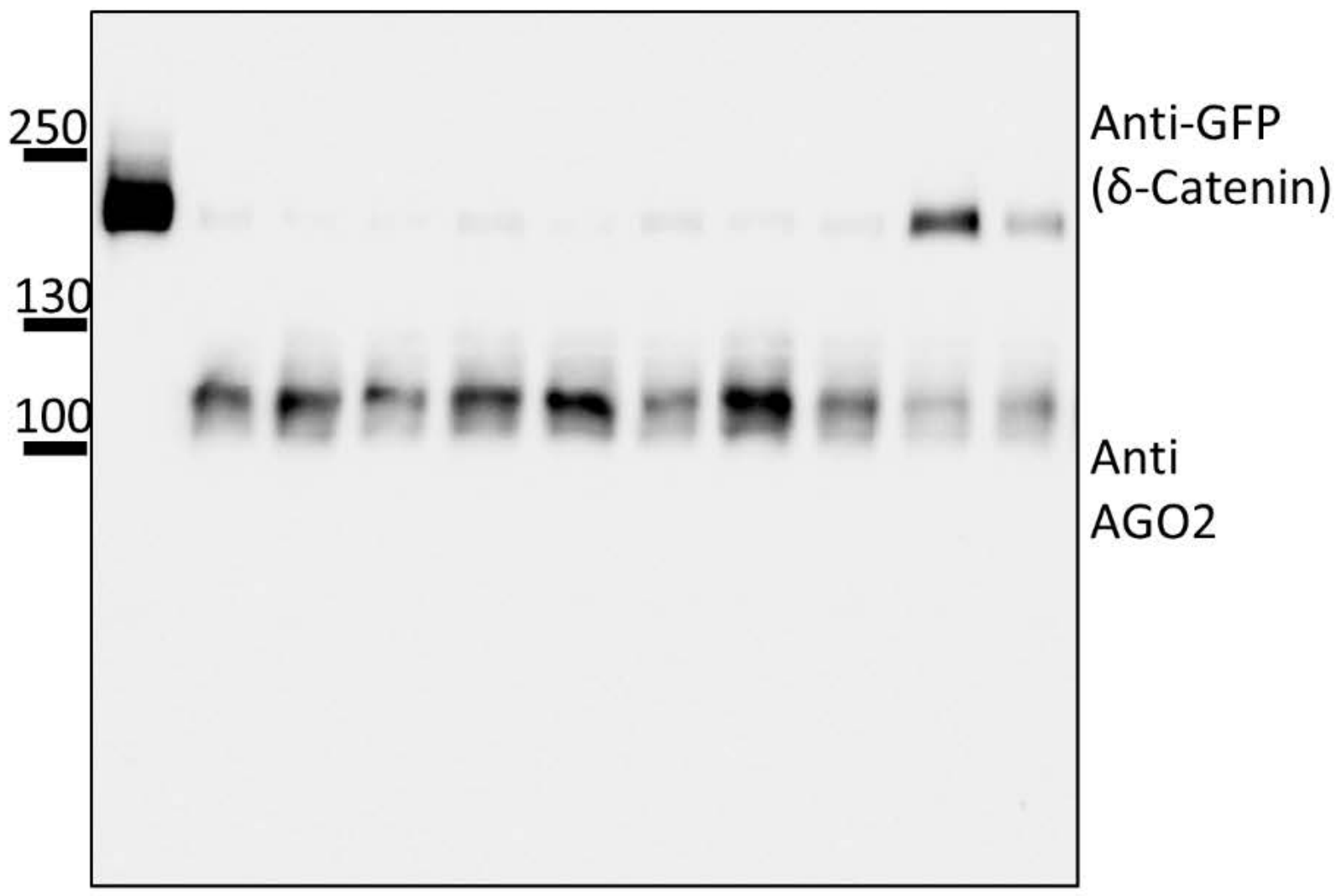

Fig. S14. Uncropped blots of Figure 2.

Uncropped blots Fig. 3a

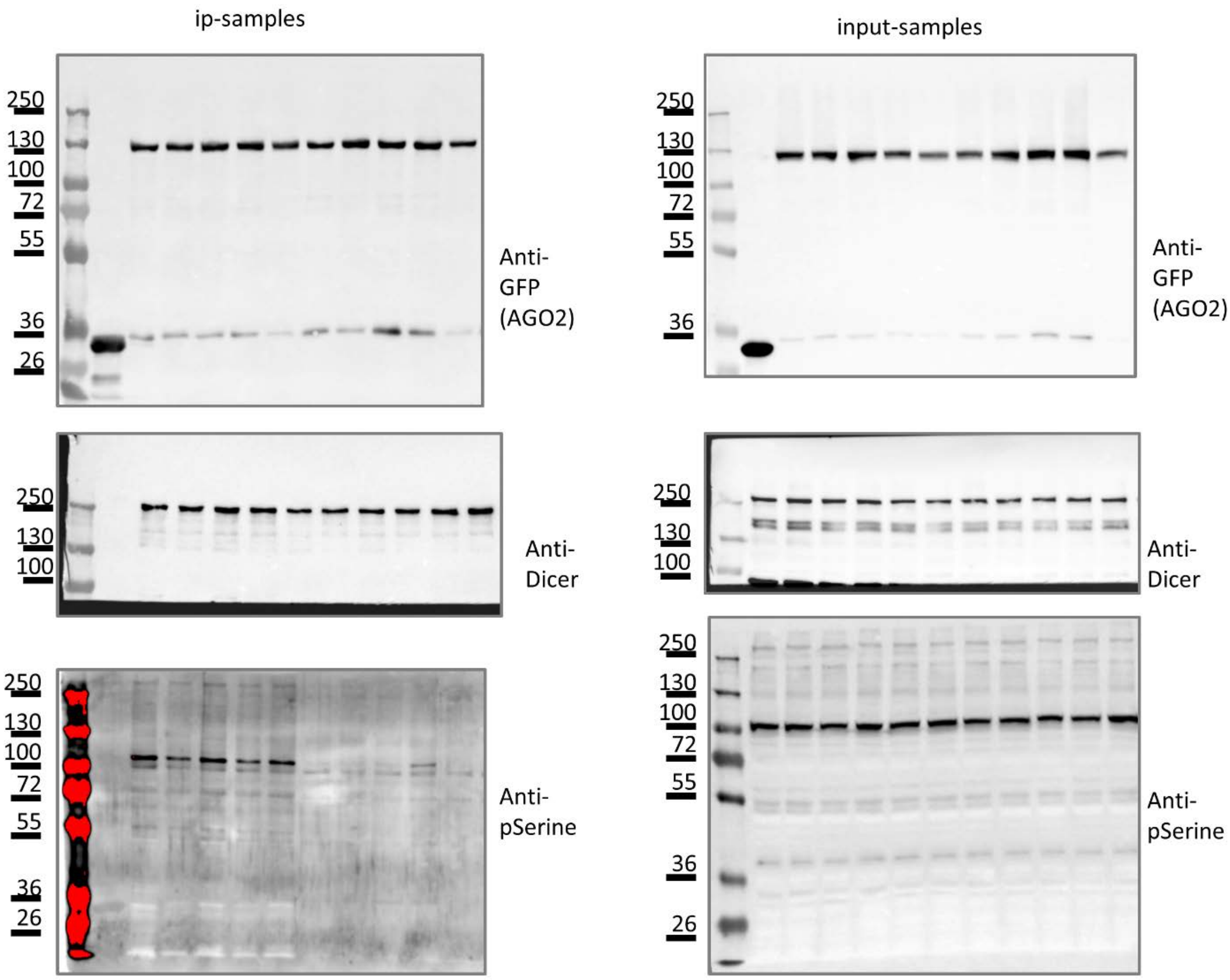

Fig. S15. Uncropped blots for Figure 3a.

Uncropped blots Fig. 3d

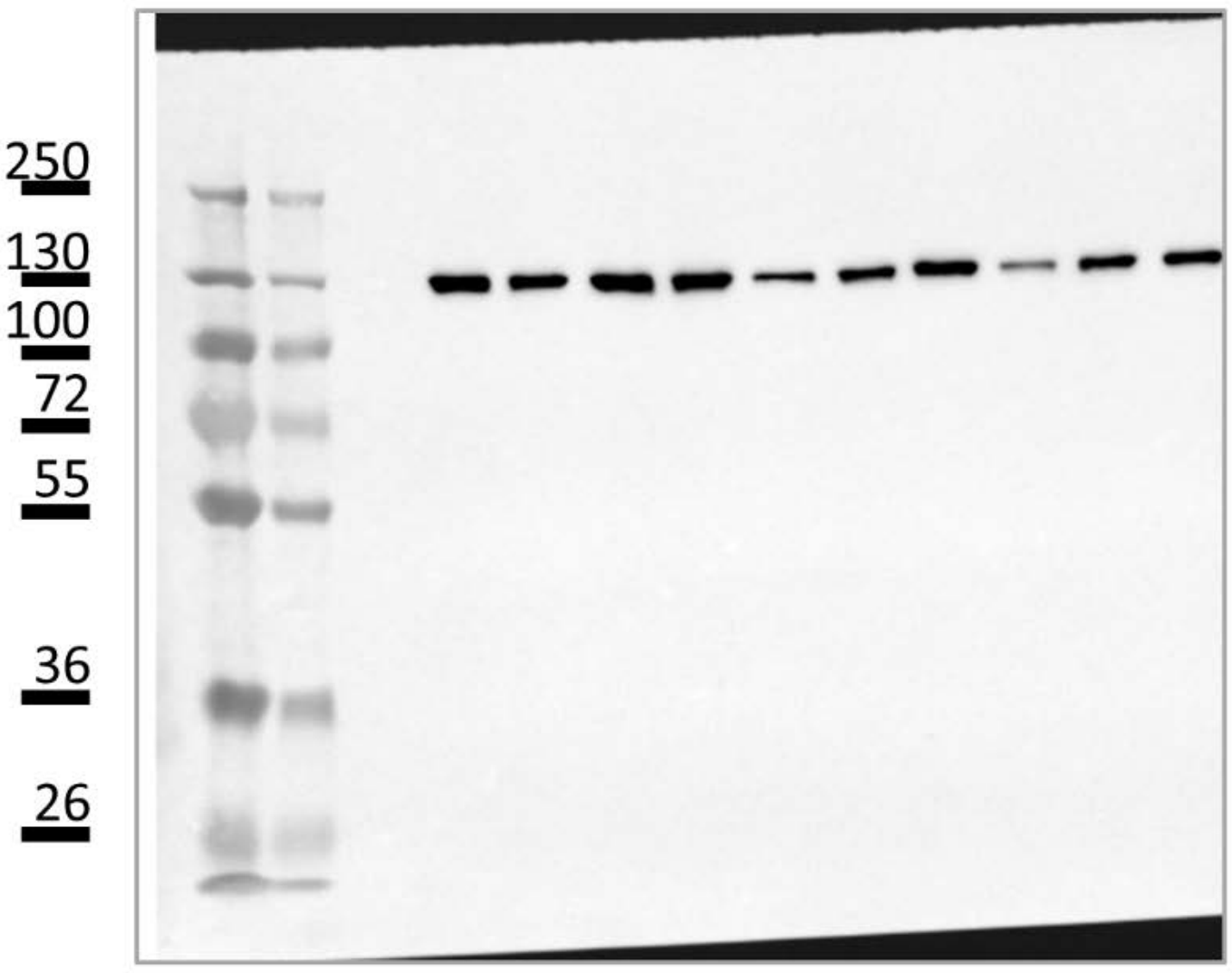

Anti-GFP
(AGO2)
IP

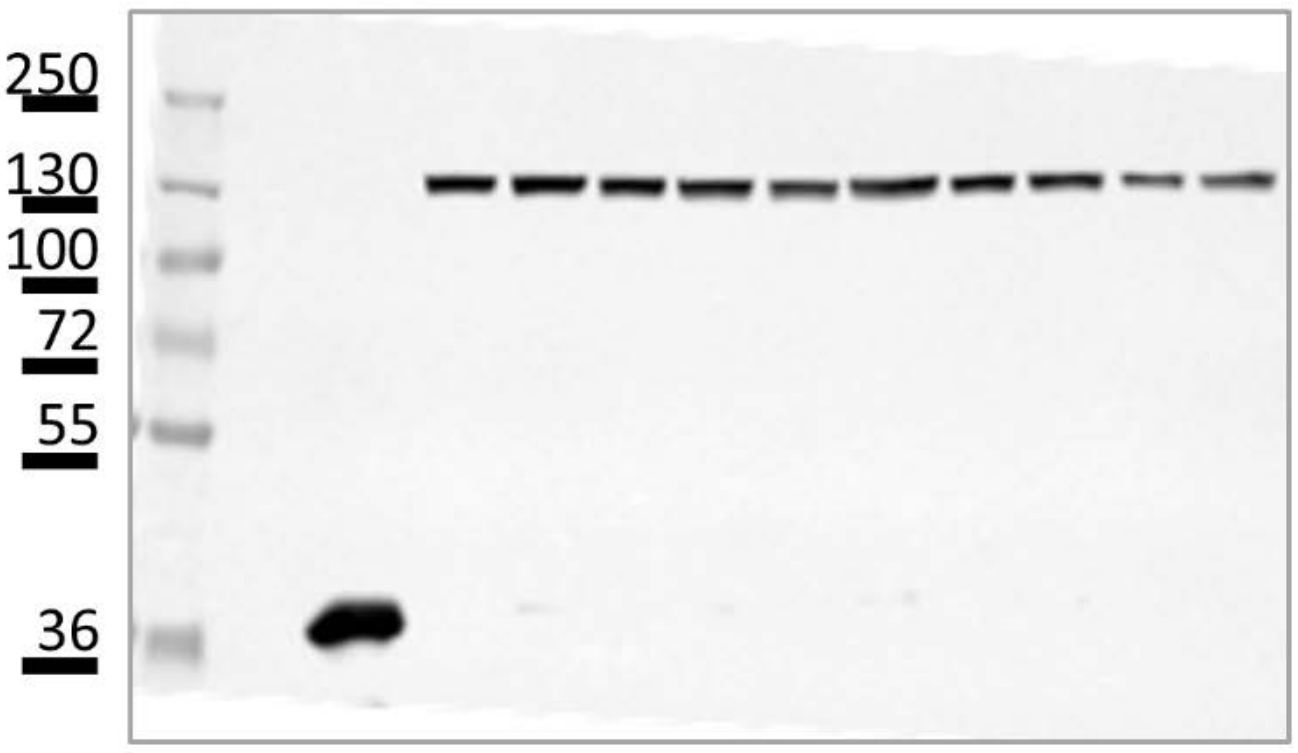

Anti-GFP
(AGO2)
IP

Fig. S16. Uncropped Blots for Figure 3d.

References.

1. Nakanishi K. Anatomy of four human Argonaute proteins. Nucleic Acids Res. 2022;50(12):6618-38.
2. Schirle NT, MacRae IJ. The crystal structure of human Argonaute2. Science. 2012;336(6084):1037-40.
